# Stochastic descriptors to study the fate and potential of naive T cell clonotypes in the periphery

**DOI:** 10.1007/s00285-016-1020-6

**Published:** 2016-06-27

**Authors:** J. R. Artalejo, A. Gómez-Corral, M. López-García, C. Molina-París

**Affiliations:** 1 Department of Statistics and Operations Research, Faculty of Mathematics, Complutense University of Madrid, 28040 Madrid, Spain; 2 Instituto de Ciencias Matemáticas, CSIC-UAM-UC3M-UCM, Calle Nicolás Cabrera 13-15, Campus de Cantoblanco UAM, 28049 Madrid, Spain; 3 Department of Applied Mathematics, School of Mathematics, University of Leeds, Leeds, LS2 9JT UK

**Keywords:** T cell homeostasis, TCR diversity, Stochastic univariate birth and death process, Extinction, Stochastic descriptor, Competition, Cross-reactivity, 60J28 Applications of continuous-time Markov processes on discrete state spaces, 92B05 General biology and biomathematics

## Abstract

The population of naive T cells in the periphery is best described by determining both its T cell receptor diversity, or number of clonotypes, and the sizes of its clonal subsets. In this paper, we make use of a previously introduced mathematical model of naive T cell homeostasis, to study the fate and potential of naive T cell clonotypes in the periphery. This is achieved by the introduction of several new stochastic descriptors for a given naive T cell clonotype, such as its maximum clonal size, the time to reach this maximum, the number of proliferation events required to reach this maximum, the rate of contraction of the clonotype during its way to extinction, as well as the time to a given number of proliferation events. Our results show that two fates can be identified for the dynamics of the clonotype: extinction in the short-term if the clonotype experiences too hostile a peripheral environment, or establishment in the periphery in the long-term. In this second case the probability mass function for the maximum clonal size is bimodal, with one mode near one and the other mode far away from it. Our model also indicates that the fate of a recent thymic emigrant (RTE) during its journey in the periphery has a clear stochastic component, where the probability of extinction cannot be neglected, even in a friendly but competitive environment. On the other hand, a greater deterministic behaviour can be expected in the potential size of the clonotype seeded by the RTE in the long-term, once it escapes extinction.

## Introduction

T cells are the set of lymphocytes characterised by the expression of a specialised receptor, called the T cell receptor (TCR). T cells can thus, be classified in “families” (or clonotypes) according to the molecular structure of the TCR they display on their membrane (Murphy et al. 2008). On average a T cell expresses $3 \times 10^4$ identical copies of a given TCR molecule. The number of T cells in the adaptive immune system of an adult human tends to a stationary “homeostatic” distribution (McLean et al. 1997; Freitas and Rocha 2000), specified by its size (the total number of T cells) and TCR diversity (the number of different clonotypes or TCR molecular structures). The size and diversity of the naive T cell pool (those T cells that have not taken part in a previous immune response) is essential to recognise a broad range of pathogens (Freitas and Rocha 1999, 2000; Tanchot and Rocha 1998). An adult mouse has a homeostatic population of $10^8$ naive T cells (Mason 1998) and a TCR diversity estimated around $2 \times 10^6$ (Zarnitsyna et al. 2013), and a healthy adult human has a homeostatic population of $10^{11}$ naive T cells (Hazenberg et al. 2003) distributed in $2.5 \times 10^7$ different TCR specificities (Zarnitsyna et al. 2013; Johnson et al. 2014).

Naive T cells originate from T cell precursors (or thymocytes) that survive positive and negative selection in the thymus (Stritesky et al. 2012; Moran and Hogquist 2012; Bird 2009). Thus, naive T cells have not been activated by exposure to pathogen-derived peptide fragments or antigens. During their lifetime, naive T cells constantly circulate in the blood to visit lymph nodes, which we refer to as the “periphery” (Takada and Jameson 2009). The population of naive T cells, which have never taken part in an immune response, is maintained by continuous, yet slow, division in the periphery (or homeostatic proliferation) (Tanchot and Rocha 1998; Troy and Shen 2003; Takada and Jameson 2009). Antigen presenting cells provide the stimulatory signals that induce naive T cell division. The signals are delivered via the interaction between TCRs (on the surface of T cells) and the ligands (also referred to as self-peptides or self-antigens) expressed on the surface of antigen presenting cells. Although the number of naive T cells is approximately constant for much of an individual’s life, as one ages both the size and the TCR diversity of the naive pool decline (Goronzy et al. 2007). This loss of T cell receptor diversity is also observed for B cells (Gibson et al. 2009), and is associated with increased susceptibility to infection and poor health. In the long-term, during the ageing process, it is important to study both the probability of extinction and the mean time to extinction for different T cell clonotypes. This clearly requires a stochastic framework. Some of the homeostatic mechanisms which ensure that a maximum of “antigen space coverage” is achieved with the available numerical diversity of T cell clonotypes (Correia-Neves et al. 2001; Mahajan et al. 2005), have been analysed mathematically before by means of deterministic (De Boer and Perelson 1997) and stochastic (Stirk et al. 2008) models. Yet, the question of how the naive T cell population remains diverse is still not completely understood (De Boer et al. 2012).

During the homeostatic period the organisation of the peripheral naive T cell pool requires that an existing T cell must die if a new T cell is generated in the thymus or by peripheral cell division (Tanchot and Rocha 1998). It is then reasonable to pose a second challenge: to quantify the probability that a given TCR specificity, namely, a single recent thymic emigrant (RTE), can be established in the periphery, while competing with the pre-existing population of naive T cells. Recent thymic emigrants are those naive T cells that have just migrated out of the thymus, after surviving positive and negative selection, to become part of the peripheral T cell population (Fink 2013). Given that double positive thymocytes hardly divide in the thymic cortex and that single positive thymocytes divide at most once in the medulla of the thymus (Sinclair et al. 2013; Stritesky et al. 2013; Sawicka et al. 2014; Yates 2014), one expects a given TCR specificity to be introduced in the periphery by a single recent thymic emigrant. There is evidence to support the fact that RTEs do not have an intrinsic lifespan (Tanchot and Rocha 1998). Given the requirement of naive T cells for continuous TCR ligation to survive in the periphery, it is natural to hypothesise that the TCR cross-reactivity of a given RTE (how many self-peptides can provide a homeostatic stimulus to the TCR under consideration) (Sewell 2012), the number of different clonotypes (competitor clonotypes) that can bind those self-peptides, and the clonal size of each competitor, are key parameters that will determine the fate and potential in the periphery of a recent thymic emigrant.

The aim of this paper is to make use of a previously introduced mathematical model of naive T cell homeostasis (Stirk et al. 2008, 2010), and define new stochastic descriptors that allow us to provide answers to the previous questions. In Refs. Stirk et al. (2008, (2010), a continuous time birth and death Markov model that describes the time evolution of a peripheral naive T cell clonotype was introduced. In this reference, it was shown that extinction takes place with certainty for all parameter values of the model, and the average time to extinction was computed. However, the question of how the affinity and the cross-reactivity of a given T cell clonotype (or RTE) determines (1) its ability to become part of the peripheral naive T cell repertoire, (2) its maximum size if the clone is to get established in the naive peripheral pool, and (3) the timescale to get established in the periphery at its maximum size, was not considered. We study these questions by defining novel stochastic descriptors for a given TCR clonotype or RTE.

The paper is organised as follows: in Sect. 2 we introduce the stochastic birth and death process and define a number of stochastic descriptors (continuous and discrete) to characterise the maximum clonal size (Sect. 2.1) and the time to reach that size, the number of divisions to reach the maximum clonal size (Sect. 2.2), and the time to get to a given number of divisions (Sect. 2.3). Section 3 provides numerical results for the previously introduced descriptors, exploring the three parameter regimes described in Sect. 2. We conclude with a discussion of our results both from a mathematical and immunological perspective in Sect. 4.

## Mathematical model

We make use of the mathematical model introduced in Stirk et al. (2008) to describe the time evolution of the number of cells in a given T cell clonotype, that is, the number of naive T cells that express identical T cell receptor molecules. The main assumption of the model is that T cells of a given clonotype compete for signals delivered by molecules (self-peptides also referred to as pMHC) expressed on antigen presenting cells (APCs). These signals induce one round of cell division, that is, a birth event. All cells of the given clonotype can also die and this is a death event in the model.

**Fig. 1:**
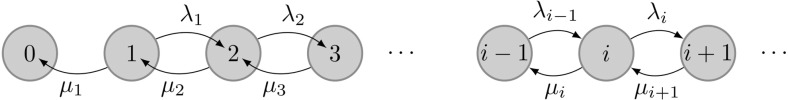
Birth and death process (with absorption) for the time evolution of the number of cells in a given naive T cell clonotype

The underlying mathematical model is a birth and death process $\{X(t):t\ge 0\}$ on the state space $\mathcal{X}=\mathbb {N}\cup \{0\}$, where the random variable *X*(*t*) describes the number of cells of a given T cell clonotype as a function of time *t*. Its birth and death rates (Fig. 1) are specified for $i \in \mathbb {N}\cup \{0\}$ by Stirk et al. (2008):

1 $$\begin{aligned} \lambda _i= & {} \varphi \; e^ {-\nu } \; \sum _{r=0}^{\infty } \; \frac{\nu ^r}{r!} \; \frac{i}{r\langle n\rangle +i}, \nonumber \\ \mu _i= & {} \mu \; i. \end{aligned}$$

Parameters $\nu \ge 0$, $\mu >0$, $\varphi >0$, and $\langle n \rangle \ge 1$ were introduced in  Stirk et al. (2008), and they have the following meaning:

- $\mu$ is the per (naive) cell death rate for cells of the clonotype under consideration,
- $\varphi$ is the per (naive) cell rate of homeostatic proliferation due to signals from the self-peptides that can bind to the TCR of the clonotype under consideration,
- $\nu$ is the number of other clonotypes (different from the one under consideration) that can compete for the same homeostatic proliferation signals, and
- $\langle n \rangle$ is the characteristic size of T cell clonotypes that compete for homeostatic proliferation signals with the clonotype of interest. We note that these competing clonotypes are not explicitly modelled and by assuming that they all have a characteristic number of naive T cells given by $\langle n \rangle$, the time evolution of the clonotype of interest can be reduced to a univariate Markov process (details about this approximation can be found in  Stirk et al. (2008)).

It was shown in  Stirk et al. (2008) that, for any value of the parameters, $0 \in \mathcal{X}$ is an absorbing state of the stochastic process, the time to extinction (or absorption) from any other state $i \in \mathcal{X}$ is finite with probability one, and its mean is also finite.

As introduced above, parameter $\varphi$ is a measure both of the affinity and the cross-reactivity of the T cell receptor for self-pMHC molecules (Sewell 2012), in the sense that the rate of homeostatic proliferation depends not only on how well a TCR can bind a given pMHC complex, but how many different pMHC complexes can provide survival signals to the T cell clonotype under consideration, characterised by its TCR molecule. Parameter $\nu$ is the number of competitors of the clonotype under consideration (Stirk et al. (2008)). Three regimes can be identified in parameter space that describe different immunological scenarios: the first one is the limit of no inter-clonal competition ($\nu \ll 1$), the second is the limit of large inter-clonal competition ($\nu \gg 1$), and the third one is the intermediate regime of competition ($\nu \approx 1$).

*Hard niche clonotype* In the special case $\nu \ll 1$, the population under study (the number of T cells that belong to a given TCR clonotype) does not compete with any other clonotypes, and thus the previous expressions for the birth and death rates simplify to (Stirk et al. (2008))

$$\begin{aligned} \lambda _i= & {} \varphi , \\ \mu _i= & {} \mu \; i. \end{aligned}$$

*Soft niche clonotype* The opposite limit, $\nu \gg 1$, represents a highly competitive environment, where the population of T cells under study competes with a large number of different clonotypes, and thus the expressions for the birth and death rates become (Stirk et al. (2008))

$$\begin{aligned} \lambda _i= & {} \frac{\varphi \; i}{ \nu \; \langle n \rangle +i}, \\ \mu _i= & {} \mu \; i. \end{aligned}$$

*Intermediate niche clonotype* In the case $\nu \approx 1$, the TCR clonotype will be referred to as an intermediate niche clonotype, and its birth and death rates will be given by Eq. (1).

These three regimes, hard, soft and intermediate niche clonotypes, will be explored in Sect. 3. Under these regimes, we introduce in this Section several stochastic descriptors which will allow us to study the dynamics of the clonotype under consideration. In particular, in Sect. 2.1 our interest is in the maximum clonal size reached by the clonotype and the time to reach this maximum size, for which we obtain analytical expressions for its probability mass function and its different order moments, respectively. We analyse the number of proliferation events to reach the maximum clonal size in Sect. 2.2, and in Sect. 2.3 we focus on the time to reach a given number of proliferation (or division) events, as a measure of the proliferative capacity (or potential) of the clonotype under study. Finally, the rate of contraction of the clonotype during its way to extinction can be studied by means of the time to contraction to a given clonal size, which is also analysed in Sect. 2.1.

### Maximum clonal size and time to reach it

Our interest in this section is in the maximum number of cells belonging to the clonotype under consideration and the time needed to reach this maximum. To begin with, we define the random variables

$$\begin{aligned} X_i^{max}= & {} \max \{X(t): t\ge 0 | X(0)=i\}, \\ T_i^{max}= & {} \inf \{t: X(t)=X_i^{max}\}, \end{aligned}$$

for $i\in \mathcal{X}$, which amount to the maximum clonal size attained by the clonotype, under the assumption that the initial size equals *i*, and the time to reach this maximum clonal size, respectively; note that $X_0^{max}=0$ and $T_0^{max}=0$, since 0 is an absorbing state in $\mathcal{X}$. In order to study both variables in parallel, we define the time to reach a total clonal size $i_{\varrho }$, given that the initial clonal size equals *i*, as the auxiliary random variable

$$\begin{aligned} T_{i,i_{\varrho }}= & {} \inf \{t: X(t)=i_{\varrho } | X(0)=i\}, \end{aligned}$$

for $i,i_{\varrho }\in \mathcal{X}$, with $T_{i_{\varrho },i_{\varrho }}=0$. We point out here that these auxiliary random variables have their own immunological interest, since attaining a given clonal size $i_{\varrho }$ might indicate that the clonotype has become large enough to mount an immune response in a timely fashion, or it has grown too large and might lead to autoimmunity, so that a tolerant immune state has been lost. The analysis of $T_{i,i_{\varrho }}$ also allows the study of the random variables of interest $X_i^{max}$ and $T_i^{max}$.

For a particular clonal size $i_{\varrho }\in \mathcal{X}$, the random variable $T_{i,i_{\varrho }}$ might be analysed in a different manner depending on whether $i_{\varrho }<i$ (time to *contraction* to a given clonal size $i_{\varrho }$), or $i_{\varrho }>i$ (time to *expansion* to a given clonal size $i_{\varrho }$). We note that in the latter case, reaching $i_{\varrho }$ is not certain, so that $T_{i,i_{\varrho }}$ is a defective random variable, while reaching $i_{\varrho }$ is certain in the former case, since absorption at state 0 occurs with probability one (Stirk et al. 2008).

#### Time to contraction to a given clonal size $i_{\varrho }<i$, given the initial clonal size *i*

For an initial clonal size *i* with $i>i_{\varrho }$, the random variable $T_{i,i_{\varrho }}$ amounts to the time until absorption into $i_{\varrho }$ for a birth and death process defined on a single absorbing state $i_{\varrho }$ and the class of transient states, $\{i_{\varrho }+1,i_{\varrho }+2,\dots \}$, with birth rates $\{\lambda _j:j\ge i_{\varrho }+1\}$ and death rates $\{\mu _j:j\ge i_{\varrho }+1\}$. Since absorption of the underlying process $\{X(t):t\ge 0\}$ occurs in a finite time almost surely (see  Stirk et al. (2008)), an appeal to  Karlin and McGregor (1957) allows us to describe the expected values of $T_{i,i_{\varrho }}$ from the equality

$$\begin{aligned} E\left[ T_{i,i_{\varrho }}^k\right]= & {} k \; \sum \limits _{n=i_{\varrho }}^{i-1} \; \rho _n \; \sum \limits _{j=n+1}^{\infty } \; \frac{E\left[ T_{j,i_{\varrho }}^{k-1}\right] }{\lambda _j \; \rho _j},\quad k\ge 1, \end{aligned}$$

where $\rho _{i_{\varrho }}=1$ and $\rho _n=\prod _{k=i_{\varrho }+1}^n\lambda _k^{-1}\mu _k$, for $n\ge i_{\varrho }+1$; note that the expected values $E[T_{i,0}^k]$ corresponding to $i_{\varrho }=0$ are related to the total extinction of the clonotype. We refer the reader to  Artalejo et al. (2012), where an analogous descriptor is analysed for the spread of an SIS epidemic among a population consisting of *N* individuals. In this case, the series $\sum _{j=n+1}^{\infty }$ becomes the finite sum $\sum _{j=n+1}^{N}$ and $T_{i,i_{\varrho }}$ can be seen as a phase-type random variable (see e.g.,  [Kulkarni (1996), Sect. 6.7] and  [Latouche and Ramaswami (1999), Chapter 2]).

#### Time to expansion to a given clonal size $i_{\varrho }>i$, given the initial clonal size *i*, and analysis of $X_i^{max}$ and $T_i^{max}$

For a fixed (and given) clonal size $i_{\varrho }\in \mathbb {N}$, we introduce the following notation:

2 $$\begin{aligned} v_{i,i_{\varrho }}= & {} {\mathbb P}(T_{i,i_{\varrho }}< \infty ) \ = \ {\mathbb P}(X_{i}^{max}\ge i_{\varrho }), \quad 0\le i\le i_{\varrho },\nonumber \\ \phi _{i,i_{\varrho }} (s)= & {} E \left[ e^{-s \; T_{i,i_{\varrho }}} \; 1_{\{T_{i,i_{\varrho }}< \infty \}} \right] , \quad 0\le i\le i_{\varrho }, \quad \mathfrak {R}(s) \ge 0,\\ m_{i,i_{\varrho }}^{(k)}= & {} E \left[ \left( T_{i,i_{\varrho }}\right) ^k 1_{\{T_{i,i_{\varrho }} < \infty \}} \right] \ = \ \left. (-1)^k\frac{d^k}{ds^k}\phi _{i,i_{\varrho }}(s)\right| _{s=0} , \quad 0\le i\le i_{\varrho },\quad k\ge 0,\nonumber \end{aligned}$$

where $1_{\{T_{i,i_{\varrho }}<\infty \}}$ is a random variable that takes the values 1 if $T_{i,i_{\varrho }}<\infty$, and 0 otherwise. The quantities defined in Eq. (2) will allow us to analyse the random variables $X_i^{max}$ and $T_i^{max}$, but the distribution of $T_{i,i_{\varrho }}$ is defective, since $T_{i,i_{\varrho }}=\infty$ if the clonotype becomes extinct before reaching the size $i_{\varrho }$; note that $1-v_{i,i_{\varrho }}$ is the probability of such an extinction event, as

$$\begin{aligned} v_{i,i_{\varrho }}= & {} \left\{ \begin{array}{ll} 0, &{}\quad \hbox {if}\,\, i=0, \\ 1, &{} \quad \hbox {if}\,\, i=i_{\varrho }, \end{array}\right. \end{aligned}$$

with $v_{i,i_{\varrho }}\in (0,1)$ in the case $1\le i\le i_{\varrho }-1$.

By a first-step argument, the restricted Laplace-Stieltjes transforms satisfy the following set of linear equations:

3 $$\begin{aligned} \phi _{0,i_{\varrho }}(s)= & {} 0 ,\nonumber \\ \phi _{i,i_{\varrho }}(s)= & {} \frac{\mu _i}{s+\lambda _i+\mu _i} \; \phi _{i-1,i_{\varrho }}(s) + \frac{\lambda _i}{s+\lambda _i+\mu _i} \; \phi _{i+1,i_{\varrho }}(s), \quad 1 \le i \le i_{\varrho }-1,\nonumber \\ \phi _{i_{\varrho },i_{\varrho }}(s)= & {} 1. \end{aligned}$$

These equations can be rewritten in multiplicative form as

$$\begin{aligned} \beta _i \; \phi _{i-1,i_{\varrho }}(s) + \gamma _i \; \phi _{i,i_{\varrho }}(s) + \alpha _i\; \phi _{i+1,i_{\varrho }}(s)= & {} \delta _i, \quad 1 \le i \le i_{\varrho }-1 \; , \end{aligned}$$

with

$$\begin{aligned} \alpha _i= & {} \left\{ \begin{array}{ll} -\lambda _i, &{} \quad \hbox {if}\quad 1\le i\le i_{\varrho }-2, \\ 0, &{} \quad \hbox {if}\quad i=i_{\varrho }-1, \end{array}\right. \\ \beta _i= & {} \left\{ \begin{array}{ll} 0, &{}\quad \hbox {if}\quad i=1, \\ -\mu _i, &{} \quad \hbox {if} \quad 2\le i\le i_{\varrho }-1, \end{array}\right. \\ \gamma _i= & {} s+\lambda _i+\mu _i,\quad 1\le i\le i_{\varrho }-1, \\ \delta _i= & {} \left\{ \begin{array}{ll} 0, &{} \quad \hbox {if}\quad 1\le i\le i_{\varrho }-2, \\ \lambda _{i_{\varrho }-1}, &{}\quad \hbox {if}\quad i=i_{\varrho }-1. \end{array}\right. \end{aligned}$$

Then, by using forward elimination, we may obtain

$$\begin{aligned} G_i \; \phi _{i,i_{\varrho }}(s) + \alpha _i\; \phi _{i+1,i_{\varrho }}(s)= & {} D_i, \quad 1 \le i \le i_{\varrho }-1, \end{aligned}$$

with

$$\begin{aligned} G_i= & {} \left\{ \begin{array}{ll} \gamma _1, &{} \quad \hbox {if}\,\,i=1, \\ \gamma _i-\frac{\beta _i \; \alpha _{i-1}}{G_{i-1}}, &{} \quad \hbox {if}\,\, 2\le i\le i_{\varrho }-1, \end{array}\right. \\ D_i= & {} \left\{ \begin{array}{ll} 0, &{} \quad \hbox {if } 1\le i\le i_{\varrho }-2, \\ \lambda _{i_{\varrho }-1}, &{} \quad \hbox {if } i=i_{\varrho }-1. \end{array}\right. \end{aligned}$$

In terms of the functions $g_i(s)=G_i-(s+\lambda _i)$, we may rewrite $\phi _{i,i_{\varrho }}(s)$ as

$$\begin{aligned} \phi _{i,i_{\varrho }}(s)= & {} \frac{D_i-\alpha _i \; \phi _{i+1,i_{\varrho }}(s)}{s+\lambda _i+g_i(s)}, \end{aligned}$$

since $$g_i(s)$$ satisfies

$$\begin{aligned} g_i(s)= & {} \mu _i \; \frac{s+g_{i-1}(s)}{s+\lambda _{i-1}+g_{i-1}(s)},\quad 2\le i\le i_{\varrho }-1, \end{aligned}$$

with $g_1(s)=\mu _1$. This implies that

4 $$\begin{aligned} \phi _{i,i_{\varrho }}(s)= & {} \prod _{k=i}^{i_{\varrho }-1}\frac{\lambda _k}{s+g_k(s)+\lambda _k},\quad 1\le i\le i_{\varrho }-1, \end{aligned}$$

from which it follows that $v_{i,i_{\varrho }}\in (0,1)$ for clonal sizes $1\le i\le i_{\varrho }-1$, since $v_{i,i_{\varrho }}=\phi _{i,i_{\varrho }}(0)$.

In evaluating the *restricted* moments $m^{(k)}_{i,i_{\varrho }}$, we first derive the probabilities $v_{i,i_{\varrho }}$ as the values $\phi _{i,i_{\varrho }}(0)$ from Eq. (4). In particular, it is seen that $v_{0,i_{\varrho }}=0$ and we can write

$$\begin{aligned} v_{i,i_{\varrho }}= & {} \left( \sum _{m=0}^{i_{\varrho }-1}\zeta _m \right) ^{-1}\sum _{k=0}^{i-1} \; \zeta _k,\quad 1\le i\le i_{\varrho }, \end{aligned}$$

where $\zeta _0=1$ and $\zeta _i=\prod _{k=1}^{i}\lambda ^{-1}_k\mu _k$, for $1\le i\le i_{\varrho }-1$. Then, we have

$$\begin{aligned} m^{(0)}_{i,i_{\varrho }}= & {} v_{i,i_{\varrho }},\quad 0\le i\le i_{\varrho }, \\ m^{(k)}_{0,i_{\varrho }}= & {} 0, \quad k\ge 1, \\ m^{(k)}_{i_{\varrho },i_{\varrho }}= & {} 0, \quad k\ge 1. \end{aligned}$$

Moments $m^{(k)}_{i,i_{\varrho }}$, for clonal sizes $1\le i\le i_{\varrho }-1$, are derived from Eq. (3). In fact, by taking derivatives in Eq. (3) we obtain

5 $$\begin{aligned} ({\lambda _i+\mu _i}) \; m_{i,i_{\varrho }}^{(k)}= & {} \mu _i \; m_{i-1,i_{\varrho }}^{(k)} + \lambda _i \; m_{i+1,i_{\varrho }}^{(k)} + k \; m_{i,i_{\varrho }}^{(k-1)}, \quad 1 \le i \le i_{\varrho }-1, \quad k \ge 1 \;.\nonumber \\ \end{aligned}$$

In order to solve Eq. (5), for a fixed value $k \ge 1$, we introduce some notation as follows:

$$\begin{aligned} x_{i,i_{\varrho }}= & {} m_{i,i_{\varrho }}^{(k)}, \\ \xi _{i,i_{\varrho }}= & {} k \; m_{i,i_{\varrho }}^{(k-1)}, \, \quad 0 \le i \le i_{\varrho }\; , \\ y_{i,i_{\varrho }}= & {} x_{i+1,i_{\varrho }}- x_{i,i_{\varrho }}, \quad 0 \le i \le i_{\varrho }-1. \end{aligned}$$

Equation (5) is then equivalent to $\lambda _i \;y_{i,i_{\varrho }} + \xi _{i,i_{\varrho }} = \mu _i \; y_{i-1,i_{\varrho }}$, for $1 \le i \le i_{\varrho }-1$, which implies that

$$\begin{aligned} y_{i,i_{\varrho }}= & {} \zeta _i \; y_{0,i_{\varrho }} - \zeta _i \; b_i, \end{aligned}$$

where $b_i =\sum _{j=1}^{i} \frac{\xi _{j,i_{\varrho }}}{\lambda _j \; \zeta _{j}}$. As a result, for $1\le i\le i_{\varrho }-1$, we have

$$\begin{aligned} x_{i+1,i_{\varrho }}= & {} x_{1,i_{\varrho }} \sum _{j=0}^{i} \; \zeta _j - \sum _{j=1}^{i} \; \zeta _j \; b_j. \end{aligned}$$

We also have $x_{i_{\varrho },i_{\varrho }}=0$ since $m^{(k)}_{i_{\varrho },i_{\varrho }}=0$, so that

6 $$\begin{aligned} x_{1,i_{\varrho }}= & {} \frac{\sum _{j=1}^{i_{\varrho }-1} \zeta _j \; b_j}{\sum _{j=0}^{i_{\varrho }-1} \zeta _j},\nonumber \\ x_{i,i_{\varrho }}= & {} \frac{\sum _{j=i}^{i_{\varrho }-1} \zeta _j \sum _{m=0}^{i-1} \zeta _m \sum _{k=m+1}^{j} \frac{\xi _{k,i_{\varrho }}}{\lambda _k \; \zeta _k} }{\sum _{j=0}^{i_{\varrho }-1} \zeta _j},\quad 2 \le i \le i_{\varrho }-1,\\ x_{i_{\varrho },i_{\varrho }}= & {} 0.\nonumber \end{aligned}$$

Equation (6) is a recursive procedure that allows us to compute the *k*th order moments, $m_{i,i_{\varrho }}^{(k)}$, in terms of previously computed moments, $m_{i,i_{\varrho }}^{(k-1)}$, since $x_{i,i_{\varrho }}=m_{i,i_{\varrho }}^{(k)}$ and $\xi _{i,i_{\varrho }}=k \; m_{i,i_{\varrho }}^{(k-1)}$. In the special cases $k=0$ and 1, it is readily seen that

$$\begin{aligned} \lim _{i_{\varrho }\rightarrow \infty }v_{i,i_{\varrho }}= & {} \left\{ \begin{array}{ll} 0, &{} \quad \hbox {if}\,\,0\le i\le i_{\varrho }-1, \\ 1, &{} \quad \hbox {if}\,\, i=i_{\varrho }, \end{array}\right. \end{aligned}$$

and the asymptotic value $\lim _{i_{\varrho }\rightarrow \infty }m_{i,i_{\varrho }}^{(1)}$ is always finite, with $\lim _{i_{\varrho }\rightarrow \infty }m_{i,i_{\varrho }}^{(1)}=0$ for initial clonal sizes $i\in \{0,i_{\varrho }\}$, since $\sum _{m=0}^{\infty }\zeta _m =\infty$, for any value of the parameter $\nu$.

We finally focus on the random variables $X_i^{max}$ and $T_i^{max}$. Note that the distribution of $X_i^{max}$ is readily derived from the values $v_{i,i_{\varrho }}$, since $v_{i,i_{\varrho }}=\mathbb {P}(X_i^{max}\ge i_{\varrho })$, and the *k*th order moment, $m_i^{max,(k)}$, of the time $T_i^{max}$ to reach the maximum clonal size can be evaluated as

$$\begin{aligned} m_i^{max,(k)}= & {} \sum _{i_{\varrho }=i}^{\infty }E\left[ \left( T_i^{max}\right) ^k\left| X_i^{max}=i_{\varrho }\right. \right] \mathbb {P}(X_i^{max}=i_{\varrho }) \\= & {} \sum _{i_{\varrho }=i+1}^{\infty } m^{(k)}_{i,i_{\varrho }}\left( 1-\frac{v_{i,i_{\varrho }+1}}{v_{i,i_{\varrho }}}\right) . \end{aligned}$$

For practical or computational purposes, the above series should be replaced by the finite sum

7 $$\begin{aligned} \sum _{i_{\varrho }=i+1}^{K_q} m^{(k)}_{i,i_{\varrho }}\left( 1-\frac{v_{i,i_{\varrho }+1}}{v_{i,i_{\varrho }}}\right) , \end{aligned}$$

where $K_q$ can be selected as the (100*q*)th percentile of $X_i^{max}$ for a probability $q\in (0,1)$ close enough to 1. Although an analytical study of $\sum _{i_{\varrho }=K_q+1}^{\infty } m^{(k)}_{i,i_{\varrho }}(1-v_{i,i_{\varrho }}^{-1}v_{i,i_{\varrho }+1})$ does not seem to be feasible, we note that by using the finite sum in Eq. (7) we ensure that the probability mass accumulated by $X_i^{max}$ is greater than *q*, whence the probability $1-q$ can be interpreted as a global error measure. Furthermore, this type of truncation procedure has been efficiently used for epidemics (Almaraz et al. 2016) and competition processes (Gómez-Corral and López García 2011, 2012).

### Number of proliferation events to reach the maximum clonal size

We are now interested in the number $N_i^{max}$ of one-step transitions $i'\rightarrow i'+1$ of $\mathcal{X}$ occurring in the random interval $[0,T_i^{max}]$, which allows us to record the number of proliferation events to reach the maximum clonal size, for $i\in \mathcal{X}$. This descriptor can be seen as a discrete version of the random variable $T_i^{max}$ in Sect. 2.1, and its analysis can be carried out by means of the number of proliferation events to reach a total clonal size, which is defined as the number $N_{i,i_{\varrho }}$ of one-step transitions $i'\rightarrow i'+1$ of $\mathcal{X}$ to register $X(t)=i_{\varrho }$ for the first time.

We first introduce the following notation:

$$\begin{aligned} \psi _{i,i_{\varrho }}(z)= & {} E\left[ z^{N_{i,i_{\varrho }}} \; 1_{\{N_{i,i_{\varrho }}<\infty \}}\right] ,\quad |z|\le 1, \\ u_{i,i_{\varrho }}= & {} \mathbb {P}(N_{i,i_{\varrho }}<\infty ), \\ \tilde{m}^{(k)}_{i,i_{\varrho }}= & {} E\left[ N_{i,i_{\varrho }}(N_{i,i_{\varrho }}-1) \cdots (N_{i,i_{\varrho }}-k+1)1_{\{N_{i,i_{\varrho }}<\infty \}} \right] ,\quad k\ge 0, \end{aligned}$$

for initial clonal sizes $1\le i\le i_{\varrho }$. It can be easily verified that $\psi _{0,i_{\varrho }}(z)=u_{0,i_{\varrho }}=\tilde{m}^{(k)}_{0,i_{\varrho }}=0$, $\psi _{i_{\varrho },i_{\varrho }}(z)=u_{i_{\varrho },i_{\varrho }}=1$ and $\tilde{m}^{(k)}_{i_{\varrho },i_{\varrho }}=0$, for $k\ge 1$. For clonal sizes $1\le i\le i_{\varrho }-1$, it is seen that $u_{i,i_{\varrho }}=v_{i,i_{\varrho }}$, since $\psi _{i,i_{\varrho }}(1)=\phi _{i,i_{\varrho }}(0)$ and, consequently, the probabilities $u_{i,i_{\varrho }}$ can be computed from Eq. (4), and they amount to the probabilities of reaching the clonal size $i_{\varrho }$ before extinction, given that the initial clonal size is *i*.

By taking derivatives on the equality

$$\begin{aligned} \psi _{i,i_{\varrho }}(z)= & {} \frac{\mu _i}{\lambda _i+\mu _i} \; \psi _{i-1,i_{\varrho }}(z) + \frac{\lambda _i}{\lambda _i+\mu _i} \; z \; \psi _{i+1,i_{\varrho }}(z), \quad 1 \le i \le i_{\varrho }-1, \end{aligned}$$

we obtain

8 $$\begin{aligned} (\lambda _i + \mu _i) \; {\tilde{m}}_{i,i_{\varrho }}^{(k)}= & {} \mu _i \; {\tilde{m}}_{i-1,i_{\varrho }}^{(k)} + \lambda _i \; {\tilde{m}}_{i+1,i_{\varrho }}^{(k)} + \lambda _i \; k \; {\tilde{m}}_{i+1,i_{\varrho }}^{(k-1)}, \quad 1 \le i \le i_{\varrho }-1, \quad k \ge 1,\nonumber \\ \end{aligned}$$

which is similar to Eq. (5) with the term $k \; m_{i,i_{\varrho }}^{(k-1)}$ replaced by $\lambda _i \; k \; {\tilde{m}}_{i+1,i_{\varrho }}^{(k-1)}$. This implies that, similarly to the solution of Eq. (6), the solution of Eq. (8) has the form

9 $$\begin{aligned} {\tilde{m}}_{i,i_{\varrho }}^{(k)}= & {} \frac{\sum _{j=i}^{i_{\varrho }-1} \zeta _j \; \sum _{m=0}^{i-1} \zeta _m \; \sum _{n=m+1}^{j} \frac{k \; {\tilde{m}}_{n+1,i_{\varrho }}^{(k-1)}}{\zeta _n} }{\sum _{j=0}^{i_{\varrho }-1} \zeta _j}, \quad 1 \le i \le i_{\varrho }-1, \quad k \ge 1.\qquad \end{aligned}$$

Similarly to Sect. 2.1.2, it can be seen that $\lim _{i_{\varrho }\rightarrow \infty }{\tilde{m}}_{i,i_{\varrho }}^{(1)}$ is always finite, with $\lim _{i_{\varrho }\rightarrow \infty }{\tilde{m}}_{i,i_{\varrho }}^{(1)}=0$ for initial clonal sizes $i\in \{0,i_{\varrho }\}$.

In the case of the random variable $N_{i,i_{\varrho }}$, not only every *k*th order factorial moment can be obtained from Eq. (9), but also its distribution. We can write

$$\begin{aligned} {\tilde{x}}_{i,i_{\varrho }}^j= & {} \mathbb P (N_{i,i_{\varrho }} = j), \quad 0 \le i \le i_{\varrho }, \quad j \ge i_{\varrho }-i, \end{aligned}$$

with ${\tilde{x}}_{0,i_{\varrho }}^j=0$ for $j\ge 0$, ${\tilde{x}}_{i_{\varrho },i_{\varrho }}^j=1$ if $j=0$, and 0 if $j\ge 1$, ${\tilde{x}}_{i,i_{\varrho }}^j=0$ for $1\le i\le i_{\varrho }-1$ and $0\le j\le i_{\varrho }-i-1$, and ${\tilde{x}}_{i,i_{\varrho }}^0=0$ for $0\le i\le i_{\varrho }-1$. For $j\ge 1$, we have

$$\begin{aligned} {\tilde{x}}_{0,i_{\varrho }}^j= & {} 0, \\ {\tilde{x}}_{i,i_{\varrho }}^j= & {} \frac{\mu _i}{\lambda _i+\mu _i} \; {\tilde{x}}_{i-1,i_{\varrho }}^j + \frac{\lambda _i}{\lambda _i+\mu _i} \; {\tilde{x}}_{i+1,i_{\varrho }}^{j-1}, \quad 1 \le i \le i_{\varrho }-1, \\ {\tilde{x}}_{i_{\varrho },i_{\varrho }}^j= & {} 0, \end{aligned}$$

so that a recursion on *j* and *i* can be obtained in order to compute the probability mass function of $N_{i,i_{\varrho }}$. The probability mass function obtained in this way is consistent with the expressions obtained in Eq. (9) for the factorial moments, but the proof is omitted here.

Finally, the factorial moments of the random variable $N_i^{max}$ can be approximated in a similar way as described for Eq. (7). In particular, if we denote ${\tilde{m}}_i^{max,(k)}=E[N_{i}^{max} (N_{i}^{max} -1) \cdots (N_{i}^{max} - k +1)]$, we have

10 $$\begin{aligned} {\tilde{m}}_i^{max,(k)}= & {} \sum \limits _{i_{\varrho }=i+1}^{\infty }{\tilde{m}}_{i,i_{\varrho }}^{(k)} \; \left( 1-\frac{v_{i,i_{\varrho }+1}}{v_{i,i_{\varrho }}} \right) \nonumber \\\approx & {} \sum \limits _{i_{\varrho }=i+1}^{K_q}{\tilde{m}}_{i,i_{\varrho }}^{(k)} \; \left( 1-\frac{v_{i,i_{\varrho }+1}}{v_{i,i_{\varrho }}} \right) . \end{aligned}$$

### Time to reach a given number of proliferation events

In this section, we fix an initial clonal size $i'$ and a number $D\ge 0$ of proliferation events, and we study the time $T_{i'}^D$ to reach a total number *D* of proliferation events (one-step transitions $i''\rightarrow i''+1$ of $\mathcal{X}$), with $T_{i'}^0=0$. In order to study this descriptor, we consider the augmented process $\{(X(t),D(t)):t\ge 0\}$, where *D*(*t*) denotes the number of division events up to time *t*, with $(X(0),D(0))=(i',0)$. For the initial clonal size $i'$, the augmented process starts from state $(i',0)$ and is defined on the finite state space

$$\begin{aligned} \mathcal{X}_0 \cup \mathcal{X}_D(i') \cup \mathcal{X}_T(i'), \end{aligned}$$

where

$$\begin{aligned} \mathcal{X}_0= & {} \{(0,d):0\le d\le D-1\},\\ \mathcal{X}_D(i')= & {} \{(i,D):2\le i\le i'+D\},\\ \mathcal{X}_T(i')= & {} \{(i,d):1\le i\le i'+d,0\le d\le D-1\}. \end{aligned}$$

**Fig. 2:**
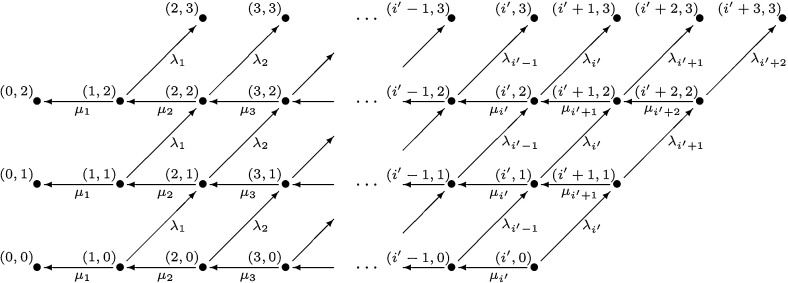
Transitions between augmented states in the case $D=3$

States in $\mathcal{X}_0\cup \mathcal{X}_D(i')$ are absorbing states. In particular, states in $\mathcal{X}_0$ represent the extinction of the clonotype when the number *D* of proliferation events has not been reached, while states in $\mathcal{X}_D(i')$ reflect that the number *D* of proliferation events has been reached. States in $\mathcal{X}_T(i')$ are transient states. Figure 2 illustrates the dynamics of the augmented process in the case $D=3$.

In a more general setting, we define the random variable

$$\begin{aligned} T^D_{(i,d)}= & {} \inf \{t: (X(t),D(t))\in \mathcal{X}_D(i') | (X(0),D(0))=(i,d)\}, \end{aligned}$$

for states $(i,d)\in \mathcal{X}_0\cup \mathcal{X}_D(i')\cup \mathcal{X}_T(i')$. Note that the descriptor $T_{i'}^D$ is equivalent to the random variable $T_{(i',0)}^D$, and states in $\mathcal{X}_0$ and $\mathcal{X}_D(i')$ lead to the values $T_{(0,d)}^D=\infty$ and $T_{(i,D)}^D=0$, respectively. For states in $\mathcal{X}_T(i')$, the random variable $T_{(i,d)}^D$ is defective, and $\mathbb {P}(T_{(i,d)}^D=\infty )>0$ is the probability of reaching $\mathcal{X}_0$ before getting to $\mathcal{X}_D(i')$.

For states $(i,d)\in \mathcal{X}_0\cup \mathcal{X}_D(i')\cup \mathcal{X}_T(i')$, we introduce the notation

$$\begin{aligned} w_{(i,d)}^D= & {} \mathbb {P}(T_{(i,d)}^D<\infty ),\\ \Phi _{(i,d)}^D(s)= & {} E\left[ e^{-sT_{(i,d)}^D} \; 1_{\{T_{(i,d)}^D<\infty \}}\right] ,\quad \mathfrak {R}(s)\ge 0,\\ {\hat{m}}_{(i,d)}^{D,(k)}= & {} E\left[ \left( T_{(i,d)}^D\right) ^k1_{\{T_{(i,d)}^D<\infty \}}\right] ,\quad k\ge 0, \end{aligned}$$

and we observe that

$$\begin{aligned} w_{(i,d)}^D= & {} \left\{ \begin{array}{ll} 0, &{} \quad if (i,d)\in \mathcal{X}_0,\\ \in (0,1), &{} \quad if (i,d)\in \mathcal{X}_T(i'),\\ 1, &{} \quad if(i,d)\in \mathcal{X}_D(i'). \end{array}\right. \end{aligned}$$

As a result, the Laplace-Stieltjes transforms $\Phi _{(i,d)}^D(s)$ verify the boundary conditions $\Phi _{(0,d)}^D(s)=0$ if $(0,d)\in \mathcal{X}_0$, and $\Phi _{(i,D)}^D (s)=1$ if $(i,D)\in \mathcal{X}_D(i')$. For states $(i,d)\in \mathcal{X}_T(i')$, we have

11 $$\begin{aligned} \Phi _{(i,d)}^D (s)= & {} \frac{\mu _i}{s+\lambda _i+\mu _i} \; \Phi _{(i-1,d)}^D(s)+\frac{\lambda _i}{s+\lambda _i+\mu _i} \; \Phi _{(i+1,d+1)}^D(s), \end{aligned}$$

which yields a finite system of linear equations that can be solved in a recursive manner (Algorithm 1).
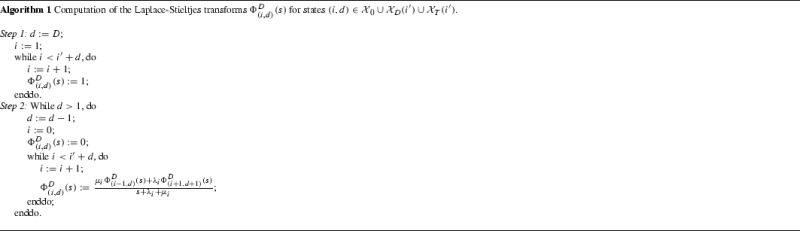


The boundary conditions for the moments ${\hat{m}}_{(i,d)}^{D,(k)}$ are given by

$$\begin{aligned} {\hat{m}}_{(i,d)}^{D,(0)}= & {} w_{(i,d)}^D,\quad (i,d)\in \mathcal{X}_0\cup \mathcal{X}_D(i')\cup \mathcal{X}_T(i'),\\ {\hat{m}}_{(i,d)}^{D,(k)}= & {} 0, \quad (i,d)\in \mathcal{X}_0\cup \mathcal{X}_D(i'), \end{aligned}$$

where the probabilities $w_{(i,d)}^D$, for states $(i,d)\in \mathcal{X}_T(i')$, are derived from Algorithm 1 by selecting $s=0$. If we take derivatives in Eq. (11), we may write down

12 $$\begin{aligned} {\hat{m}}_{(i,d)}^{D,(k)}= & {} \frac{k}{\lambda _i+\mu _i} \; {\hat{m}}_{(i,d)}^{D,(k-1)}+\frac{\mu _i}{\lambda _i+\mu _i} \; {\hat{m}}_{(i-1,d)}^{D,(k)}+\frac{\lambda _i}{\lambda _i+\mu _i} \; {\hat{m}}_{(i+1,d+1)}^{D,(k)}, \end{aligned}$$

for states $(i,d)\in \mathcal{X}^T(i')$. Eq. (12) can be solved (Algorithm 2) by adapting our arguments in Algorithm 1.
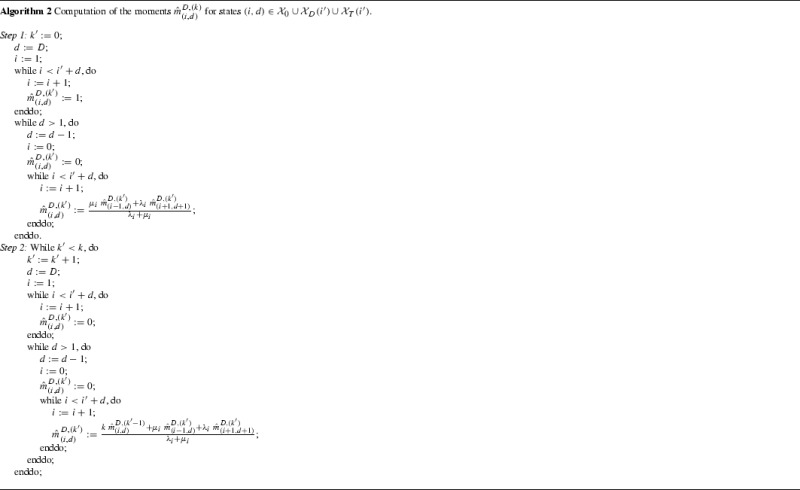


## Numerical results

In this Section we carry out a set of numerical experiments in order to analyse the potential of a recent thymic emigrant (of a given clonotype) in the periphery to expand to different clonal sizes, or to proliferate for a given number of divisions, as well as the random times of these events, and the rate of contraction of the clonotype under consideration. We study these random variables under several competition and signalling environments, which are specified by the choice of parameters $\nu ,\langle n \rangle$ and $\varphi$. We point out here that parameters $\varphi$ and $\nu$ should be seen as intrinsically related to the particular TCR expressed by the T cells within the clonotype under consideration, since this TCR determines the number of different self-peptides that the T cell can interact with. On the other hand, $\langle n \rangle$ is not directly related to the TCR and should be considered an environmental parameter, since it is the characteristic size of the competing clonotypes. We set the per cell death rate $\mu =1$, so that the time unit in the process under study is the mean lifetime of a T cell.

We consider throughout this Section homeostatic signalling rates that have been selected for a wide range of parameter values, in view of some preliminary numerical experiments. In particular, our results regarding the hard, the intermediate and the soft niche cases amount to values of the signalling rate $\varphi$, the number $\nu$ of competitors, and the characteristic size $\langle n \rangle$ of these competitors ranging from *friendly* scenarios, where the T cell clonotype under consideration can become established in the periphery, to *hostile* environments, where this establishment is not possible at all. In particular, we analyse:

**Table 1:** Expected values $E[X^{max}_i]$, $E[T^{max}_i]$ and $E[N^{max}_i]$ as a function of $\varphi$ for the hard niche case (*i.e.,* $\nu =0$), for parameters $\mu =1.0$ and $i=1$

$\varphi$	$E[X^{max}_i]$	$E[T^{max}_i]$	$E[N^{max}_i]$	*q*	0.25	0.5	0.75	0.99
0.1	1.08143	0.07834	0.08617	$K_q$	1	1	1	2
				$\mathbb {\tilde{P}}(X^{max}_i\ge K_q)$	0.99526	0.99526	0.99526	0.08617
				$E[T_{i,K_q}|X^{max}_i\ge K_q]$	0	0	0	0.90909
				$\sigma [T_{i,K_q}|X^{max}_i\ge K_q]$	0	0	0	0.90909
				$E[N_{i,K_q}|X^{max}_i\ge K_q]$	0	0	0	1
				$\sigma [N_{i,K_q}|X^{max}_i\ge K_q]$	0	0	0	0
1.0	1.84694	0.52477	1.07984	$K_q$	1	2	3	5
				$\mathbb {\tilde{P}}(X^{max}_i\ge K_q)$	0.99351	0.49351	0.24351	0.02292
				$E[T_{i,K_q}|X^{max}_i\ge K_q]$	0	0.5	1.25	2.79412
				$\sigma [T_{i,K_q}|X^{max}_i\ge K_q]$	0	0.5	1.03078	2.03962
				$E[N_{i,K_q}|X^{max}_i\ge K_q]$	0	1	2.5	6.29412
				$\sigma [N_{i,K_q}|X^{max}_i\ge K_q]$	0	0	0.86603	2.66205
5.0	8.40494	12.72341	67.15374	$K_q$	4	10	12	16
				$\mathbb {\tilde{P}}(X^{max}_i\ge K_q)$	0.74812	0.54931	0.32916	0.00999
				$E[T_{i,K_q}|X^{max}_i\ge K_q]$	0.72892	9.43564	21.56767	39.98014
				$\sigma [T_{i,K_q}|X^{max}_i\ge K_q]$	0.52755	8.08455	19.77093	37.53902
				$E[N_{i,K_q}|X^{max}_i\ge K_q]$	4.02410	49.43688	113.44516	214.03020
				$\sigma [N_{i,K_q}|X^{max}_i\ge K_q]$	1.43975	39.26485	98.40159	191.6886
10.0	21.50413	927.95677	$9.29\times 10^{3}$	$K_q$	22	24	26	29
				$\mathbb {\tilde{P}}(X^{max}_i\ge K_q)$	0.80227	0.61532	0.25744	0.01306
				$E[T_{i,K_q}|X^{max}_i\ge K_q]$	209.05940	736.272000	1745.83800	2435.43200
				$\sigma [T_{i,K_q}|X^{max}_i\ge K_q]$	206.35540	733.346400	1742.73800	2432.12500
				$E[N_{i,K_q}|X^{max}_i\ge K_q]$	$2.09\times 10^{3}$	$7.37\times 10^{3}$	$1.75\times 10^{4}$	$2.44\times 10^{4}$
				$\sigma [N_{i,K_q}|X^{max}_i\ge K_q]$	$2.06\times 10^{3}$	$7.33\times 10^{3}$	$1.74\times 10^{4}$	$2.43\times 10^{4}$
20.0	48.33950	$1.01\times 10^{7}$	$2.02\times 10^{8}$	$K_q$	50	51	53	56
				$\mathbb {\tilde{P}}(X^{max}_i\ge K_q)$	0.79967	0.65553	0.24416	0.01118
				$E[T_{i,K_q}|X^{max}_i\ge K_q]$	$3.81\times 10^{6}$	$7.71\times 10^{6}$	$1.88\times 10^{7}$	$2.51\times 10^{7}$
				$\sigma [T_{i,K_q}|X^{max}_i\ge K_q]$	$3.81\times 10^{6}$	$7.71\times 10^{6}$	$1.88\times 10^{7}$	$2.51\times 10^{7}$
				$E[N_{i,K_q}|X^{max}_i\ge K_q]$	$7.62\times 10^{7}$	$1.54\times 10^{8}$	$3.77\times 10^{8}$	$5.03\times 10^{8}$
				$\sigma [N_{i,K_q}|X^{max}_i\ge K_q]$	$7.62\times 10^{7}$	$1.54\times 10^{8}$	$3.77\times 10^{8}$	$5.03\times 10^{8}$
30.0	75.17354	$1.48\times 10^{11}$	$4.44\times 10^{12}$	$K_q$	77	78	80	83
				$\mathbb {\tilde{P}}(X^{max}_i\ge K_q)$	0.82173	0.67261	0.24499	0.01096
				$E[T_{i,K_q}|X^{max}_i\ge K_q]$	$5.25\times 10^{10}$	$1.10\times 10^{11}$	$2.73\times 10^{11}$	$3.62\times 10^{11}$
				$\sigma [T_{i,K_q}|X^{max}_i\ge K_q]$	$5.25\times 10^{10}$	$1.10\times 10^{11}$	$2.73\times 10^{11}$	$3.62\times 10^{11}$
				$E[N_{i,K_q}|X^{max}_i\ge K_q]$	$1.57\times 10^{12}$	$3.28\times 10^{12}$	$8.19\times 10^{12}$	$1.09\times 10^{13}$
				$\sigma [N_{i,K_q}|X^{max}_i\ge K_q]$	$1.57\times 10^{12}$	$3.28\times 10^{12}$	$8.19\times 10^{12}$	$1.09\times 10^{13}$
40.0	102.01704	$2.44\times 10^{15}$	$9.75\times 10^{16}$	$K_q$	104	105	107	110
				$\mathbb {\tilde{P}}(X^{max}_i\ge K_q)$	0.83761	0.69029	0.25417	0.01138
				$E[T_{i,K_q}|X^{max}_i\ge K_q]$	$8.06\times 10^{14}$	$1.72\times 10^{15}$	$4.42\times 10^{15}$	$5.93\times 10^{15}$
				$\sigma [T_{i,K_q}|X^{max}_i\ge K_q]$	$8.06\times 10^{14}$	$1.72\times 10^{15}$	$4.42\times 10^{15}$	$5.93\times 10^{15}$
				$E[N_{i,K_q}|X^{max}_i\ge K_q]$	$3.22\times 10^{16}$	$6.88\times 10^{16}$	$1.77\times 10^{17}$	$2.37\times 10^{17}$
				$\sigma [N_{i,K_q}|X^{max}_i\ge K_q]$	$3.22\times 10^{16}$	$6.88\times 10^{16}$	$1.77\times 10^{17}$	$2.37\times 10^{17}$
50.0	128.84185	$4.28\times 10^{19}$	$2.14\times 10^{21}$	$K_q$	131	132	134	137
				$\mathbb {\tilde{P}}(X^{max}_i\ge K_q)$	0.85166	0.70967	0.26879	0.01220
				$E[T_{i,K_q}|X^{max}_i\ge K_q]$	$1.30\times 10^{19}$	$2.84\times 10^{19}$	$7.60\times 10^{19}$	$1.04\times 10^{20}$
				$\sigma [T_{i,K_q}|X^{max}_i\ge K_q]$	$1.30\times 10^{19}$	$2.84\times 10^{19}$	$7.60\times 10^{19}$	$1.04\times 10^{20}$
				$E[N_{i,K_q}|X^{max}_i\ge K_q]$	$6.52\times 10^{20}$	$1.42\times 10^{21}$	$3.80\times 10^{21}$	$5.19\times 10^{21}$
				$\sigma [N_{i,K_q}|X^{max}_i\ge K_q]$	$6.52\times 10^{20}$	$1.42\times 10^{21}$	$3.80\times 10^{21}$	$5.19\times 10^{21}$
75.0	196.43319	$2.06\times 10^{30}$	$1.55\times 10^{32}$	$K_q$	199	200	201	205
				$\mathbb {\tilde{P}}(X^{max}_i\ge K_q)$	0.83576	0.67186	0.44024	0.00960
				$E[T_{i,K_q}|X^{max}_i\ge K_q]$	$7.42\times 10^{29}$	$1.58\times 10^{30}$	$2.77\times 10^{30}$	$4.97\times 10^{30}$
				$\sigma [T_{i,K_q}|X^{max}_i\ge K_q]$	$7.42\times 10^{29}$	$1.58\times 10^{30}$	$2.77\times 10^{30}$	$4.97\times 10^{30}$
				$E[N_{i,K_q}|X^{max}_i\ge K_q]$	$5.57\times 10^{31}$	$1.19\times 10^{32}$	$2.07\times 10^{32}$	$3.73\times 10^{32}$
				$\sigma [N_{i,K_q}|X^{max}_i\ge K_q]$	$5.57\times 10^{31}$	$1.19\times 10^{32}$	$2.07\times 10^{32}$	$3.73\times 10^{32}$
100.0	264.12728	$1.01\times 10^{41}$	$1.11\times 10^{43}$	$K_q$	267	268	269	273
				$\mathbb {\tilde{P}}(X^{max}_i\ge K_q)$	0.81774	0.63708	0.39927	0.00800
				$E[T_{i,K_q}|X^{max}_i\ge K_q]$	$4.59\times 10^{40}$	$9.55\times 10^{40}$	$1.61\times 10^{41}$	$2.68\times 10^{41}$
				$\sigma [T_{i,K_q}|X^{max}_i\ge K_q]$	$4.59\times 10^{40}$	$9.55\times 10^{40}$	$1.61\times 10^{41}$	$2.68\times 10^{41}$
				$E[N_{i,K_q}|X^{max}_i\ge K_q]$	$4.59\times 10^{42}$	$9.55\times 10^{42}$	$1.61\times 10^{43}$	$2.68\times 10^{43}$
				$\sigma [N_{i,K_q}|X^{max}_i\ge K_q]$	$4.59\times 10^{42}$	$9.55\times 10^{42}$	$1.61\times 10^{43}$	$2.68\times 10^{43}$

**Fig. 3:**
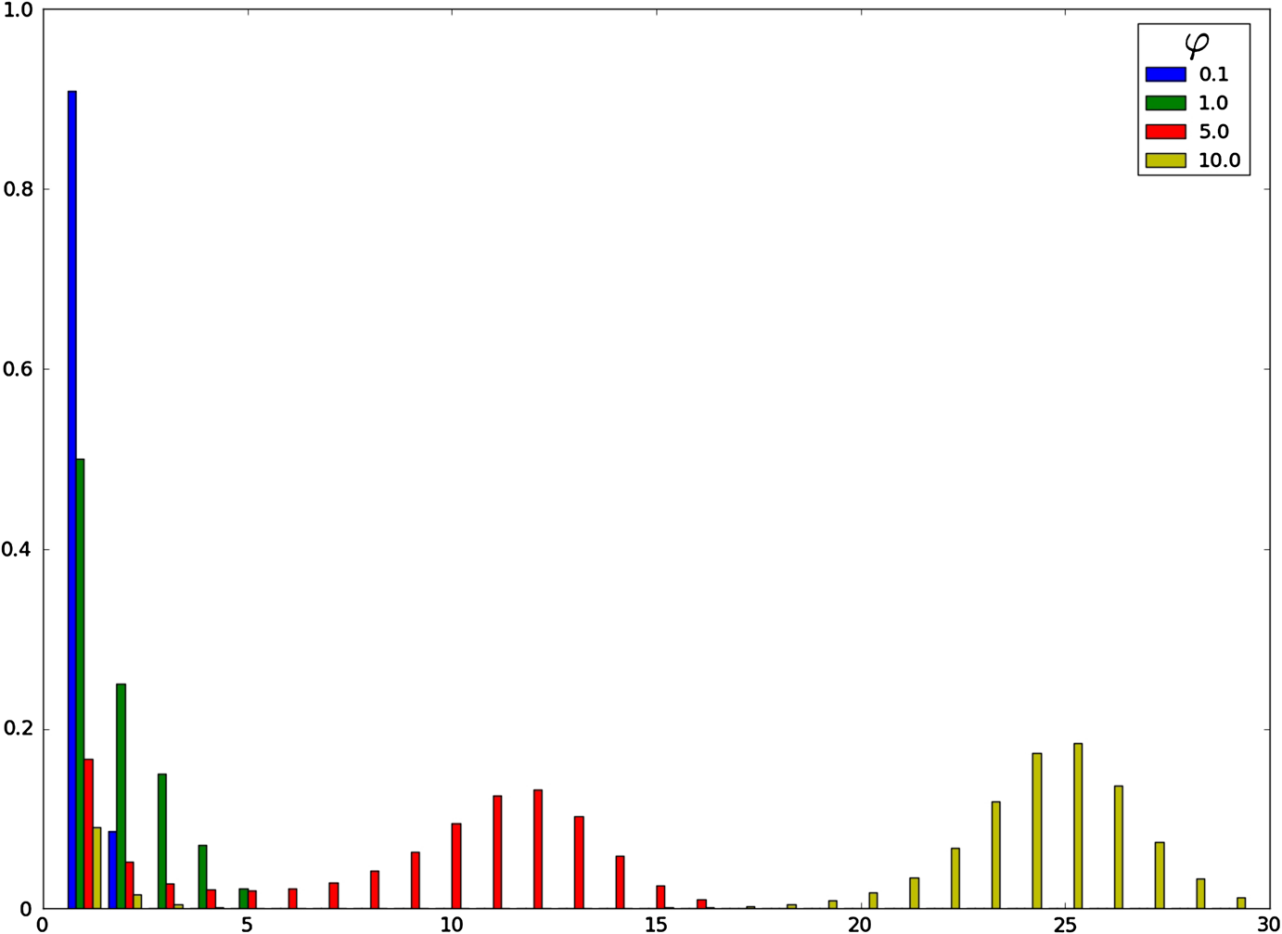
Probability mass function of $X^{max}_i$ for different values of $\varphi$ in the hard niche case (*i.e.,* $\nu =0$), for $\mu =1.0$ and the initial clonal size $i=1$

**Fig. 4:**
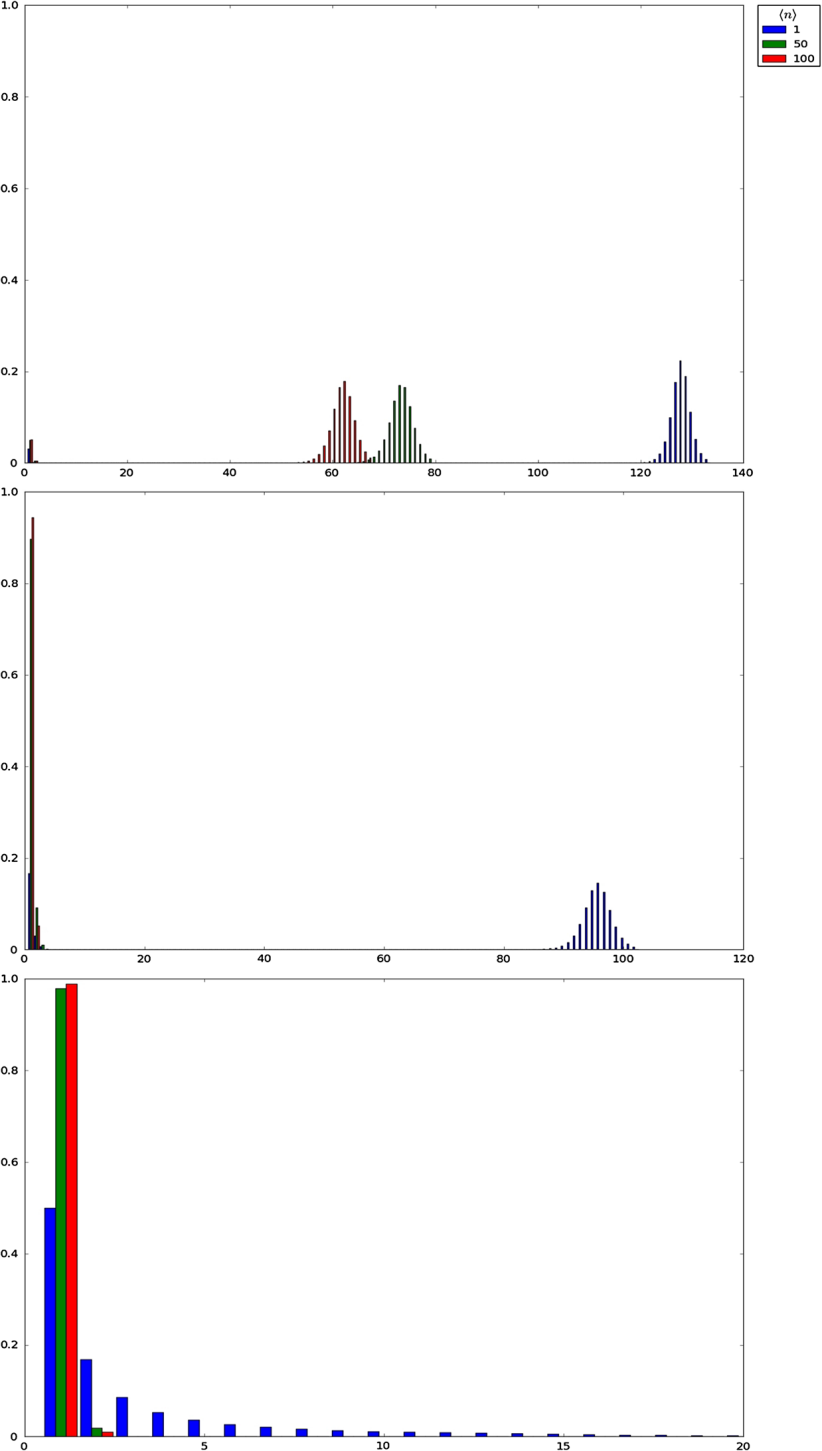
Probability mass function of $X^{max}_i$ as a function $\langle n \rangle$, in the intermediate niche case for (from *top* to *bottom*) $\nu \in \{1,10,50\}$, the parameters $(\mu ,\varphi )=(1.0,50.0)$ and the initial clonal size $i=1$

**Fig. 5:**
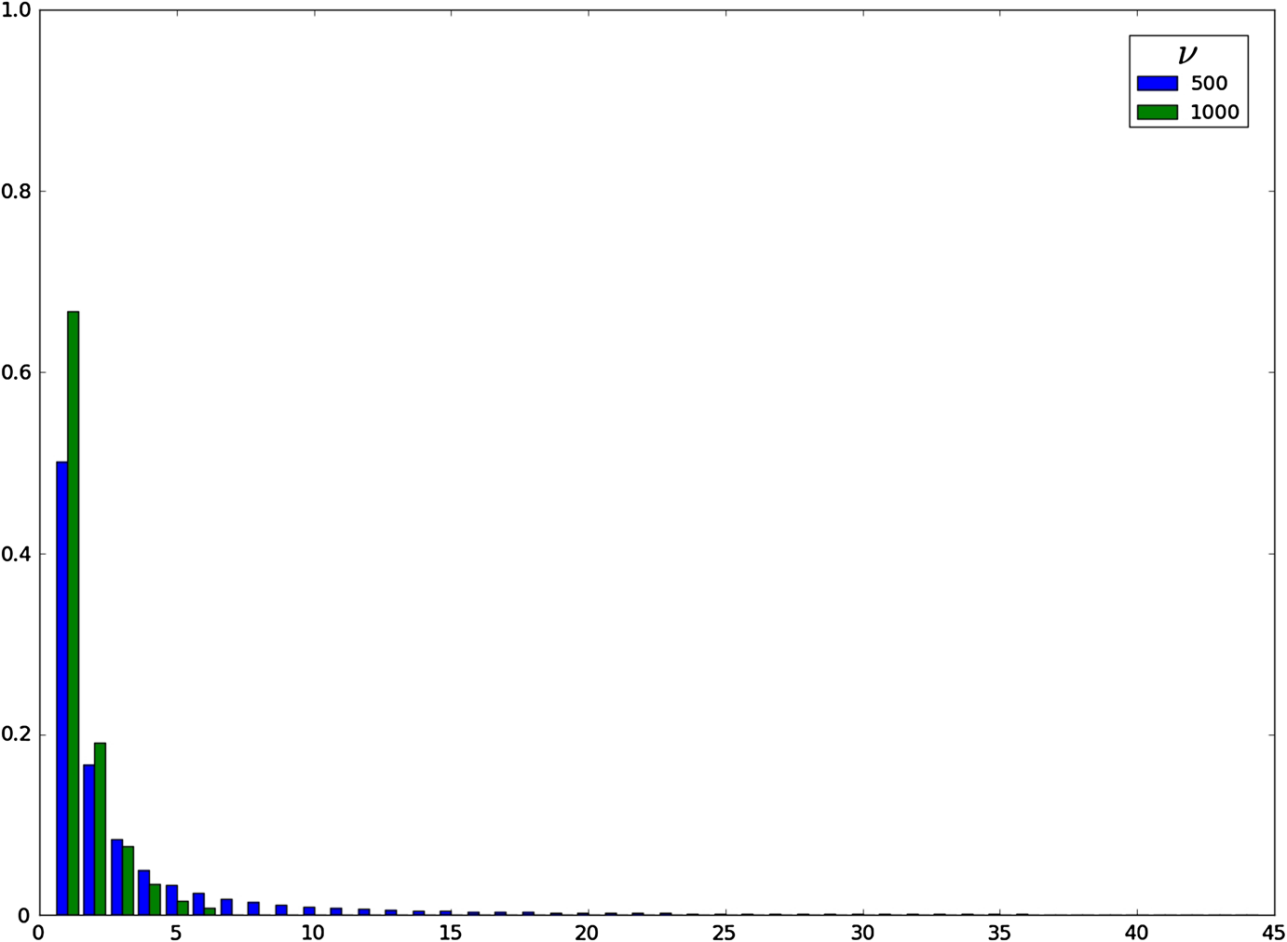
Probability mass function of $X^{max}_i$ in the soft niche case, for $\nu \in \{500,1000\}$, the parameters $(\mu ,\varphi )=(1.0,500.0)$, $\langle n \rangle =1$ and the initial clonal size $i=1$

- The hard niche case, which corresponds to a clonotype (or RTE) with no competitors ($\nu =0$), in Table 1, with numerical results based on the stochastic descriptors $X_i^{max}$, $T_i^{max}$ and $N_i^{max}$, for various small-to-moderate homeostatic signalling rates, $\varphi \in \{0.1,1.0,5.0,10.0,20.0,30.0,40.0,50.0,75.0,100.0\}$. The hard niche case is also analysed in Fig. 3, where the probability mass function of $X_i^{max}$ is plotted for various homeostatic signalling rates, $\varphi \in \{0.1,1.0,5.0,10.0\}$,
- The intermediate niche case in Table 2 and Fig. 4, which corresponds to moderate average numbers of competitors, $\nu \in \{1,10,50\}$, and moderate homeostatic signalling rates, $\varphi =50$,
- The soft niche case in Table 3 and Fig. 5, which reflects large average numbers of competitors, $\nu \in \{500,1000\}$, and high homeostatic signalling rates, $\varphi =500$.

In Tables 1, 2 and 3, the mean values $E[X_i^{max}]$, $E[T_i^{max}]$ and $E[N_i^{max}]$ are computed by considering only clonal sizes up to the 99th percentile $K_{0.99}$ of $X_i^{max}$, and by means of the truncating procedures given by Eqs. (7) and (10). However, for parameter values $(\nu ,\langle n \rangle )\in \{(500,100),(1000,50),(1000,100)\}$ in Table 3, values of $E[X_i^{max}]$, $E[T_i^{max}]$ and $E[N_i^{max}]$ are computed by considering only clonal sizes up to the percentile $K_{0.999}$ of $X_i^{max}$, since $K_{0.99}=1$ in these cases which could lead to misleading results. In this way, the quantities obtained are approximations to the *true* ones, and we focus on the particular case in which the clonotype under study has been recently released to the periphery (RTE), so that $i=1$. A direct comparison of our results with results obtained by Gillespie simulations suggests a significant level of precision for the truncation procedures proposed by Eqs. (7) and (10). For example, values $E[X_i^{max}]=8.40494$, $E[T_i^{max}]=12.72341$ and $E[N_i^{max}]=67.15374$ reported in Table 1 for $\varphi =5.0$ have Gillespie ($10^6$ simulations) counterparts $E[X_i^{max}]=8.49793$, $E[T_i^{max}]=12.91816$ and $E[N_i^{max}]=68.21512$, leading to relative error values, $1-\frac{\mathrm{truncated\,value}}{\mathrm{simulated\,value}}=0.0109$, 0.0150 and 0.0155, respectively.

**Table 2:** Expected values $E[X^{max}_i]$, $E[T^{max}_i]$ and $E[N^{max}_i]$ for different choices of $(\nu ,\langle n \rangle )$ in the intermediate niche case for the parameters $(\mu ,\varphi )=(1.0,50.0)$ and the initial clonal size $i=1$

$\nu$	$\langle n \rangle$	$E[X^{max}_i]$	$E[T^{max}_i]$	$E[N^{max}_i]$	*q*	0.25	0.5	0.75	0.99
1	1	123.25013	$$1.57\times 10^{18}$$	$$7.68\times 10^{19}$$	$$K_q $$	127	128	129	133
					$\mathbb {\tilde{P}}(X^{max}_i\ge K_q)$	0.78258	0.60668	0.38305	0.00847
					$E[T_{i,K_q}|X^{max}_i\ge K_q]$	$7.22\times 10^{17}$	$1.43\times 10^{18}$	$2.33\times 10^{18}$	$3.83\times 10^{18}$
					$\sigma [T_{i,K_q}|X^{max}_i\ge K_q]$	$7.22\times 10^{17}$	$1.43\times 10^{18}$	$2.33\times 10^{18}$	$3.83\times 10^{18}$
					$E[N_{i,K_q}|X^{max}_i\ge K_q]$	$3.54\times 10^{19}$	$7.00\times 10^{19}$	$1.14\times 10^{20}$	$1.88\times 10^{20}$
					$\sigma [N_{i,K_q}|X^{max}_i\ge K_q]$	$3.54\times 10^{19}$	$7.00\times 10^{19}$	$1.14\times 10^{20}$	$1.88\times 10^{20}$
	50	68.61117	$$2.16\times 10^{8}$$	$$5.92\times 10^{9}$$	$$K_q $$	71	73	75	79
					$\mathbb {\tilde{P}}(X^{max}_i\ge K_q)$	0.83010	0.60581	0.27023	0.00942
					$E[T_{i,K_q}|X^{max}_i\ge K_q]$	$5.97\times 10^{7}$	$1.86\times 10^{8}$	$3.75\times 10^{8}$	$5.22\times 10^{8}$
					$\sigma [T_{i,K_q}|X^{max}_i\ge K_q]$	$5.97\times 10^{7}$	$1.86\times 10^{8}$	$3.75\times 10^{8}$	$5.22\times 10^{8}$
					$E[N_{i,K_q}|X^{max}_i\ge K_q]$	$1.64\times 10^{9}$	$5.10\times 10^{9}$	$1.03\times 10^{10}$	$1.43\times 10^{10}$
					$\sigma [N_{i,K_q}|X^{max}_i\ge K_q]$	$1.64\times 10^{9}$	$5.10\times 10^{9}$	$1.03\times 10^{10}$	$1.43\times 10^{10}$
	100	57.61605	$$2.24\times 10^{7}$$	$$5.15\times 10^{8}$$	$$K_q $$	60	62	63	67
					$\mathbb {\tilde{P}}(X^{max}_i\ge K_q)$	0.78631	0.50329	0.32493	0.01130
					$E[T_{i,K_q}|X^{max}_i\ge K_q]$	$8.71\times 10^{6}$	$2.54\times 10^{7}$	$3.59\times 10^{7}$	$5.44\times 10^{7}$
					$\sigma [T_{i,K_q}|X^{max}_i\ge K_q]$	$8.71\times 10^{6}$	$2.54\times 10^{7}$	$3.59\times 10^{7}$	$5.44\times 10^{7}$
					$E[N_{i,K_q}|X^{max}_i\ge K_q]$	$2.01\times 10^{8}$	$5.85\times 10^{8}$	$8.27\times 10^{8}$	$1.25\times 10^{9}$
					$\sigma [N_{i,K_q}|X^{max}_i\ge K_q]$	$2.01\times 10^{8}$	$5.85\times 10^{8}$	$8.27\times 10^{8}$	$1.25\times 10^{9}$
10	1	75.87474	$$4.57\times 10^{9}$$	$$1.82\times 10^{11}$$	$$K_q $$	92	95	97	102
					$\mathbb {\tilde{P}}(X^{max}_i\ge K_q)$	0.75703	0.58015	0.30540	0.00584
					$E[T_{i,K_q}|X^{max}_i\ge K_q]$	$5.38\times 10^{8}$	$3.50\times 10^{9}$	$8.10\times 10^{9}$	$1.31\times 10^{10}$
					$\sigma [T_{i,K_q}|X^{max}_i\ge K_q]$	$5.38\times 10^{8}$	$3.50\times 10^{9}$	$8.10\times 10^{9}$	$1.31\times 10^{10}$
					$E[N_{i,K_q}|X^{max}_i\ge K_q]$	$2.15\times 10^{10}$	$1.40\times 10^{11}$	$3.23\times 10^{11}$	$5.24\times 10^{11}$
					$\sigma [N_{i,K_q}|X^{max}_i\ge K_q]$	$2.15\times 10^{10}$	$1.40\times 10^{11}$	$3.23\times 10^{11}$	$5.24\times 10^{11}$
	50	1.11079	0.09735	0.11315	$$K_q $$	1	1	1	3
					$\mathbb {\tilde{P}}(X^{max}_i\ge K_q)$	0.99869	0.99869	0.99869	0.01028
					$E[T_{i,K_q}|X^{max}_i\ge K_q]$	0	0	0	1.48326
					$\sigma [T_{i,K_q}|X^{max}_i\ge K_q]$	0	0	0	1.14561
					$E[N_{i,K_q}|X^{max}_i\ge K_q]$	0	0	0	2.10206
					$\sigma [N_{i,K_q}|X^{max}_i\ge K_q]$	0	0	0	0.33538
	100	1.04909	0.04937	0.05227	$$K_q $$	1	1	1	2
					$\mathbb {\tilde{P}}(X^{max}_i\ge K_q)$	0.99682	0.99682	0.99682	0.05227
					$E[T_{i,K_q}|X^{max}_i\ge K_q]$	0	0	0	0.94455
					$\sigma [T_{i,K_q}|X^{max}_i\ge K_q]$	0	0	0	0.94455
					$E[N_{i,K_q}|X^{max}_i\ge K_q]$	0	0	0	1
					$\sigma [N_{i,K_q}|X^{max}_i\ge K_q]$	0	0	0	0
50	1	2.99647	1.06499	4.53753	$$K_q $$	1	2	3	20
					$\mathbb {\tilde{P}}(X^{max}_i\ge K_q)$	0.99229	0.49229	0.32338	0.00242
					$E[T_{i,K_q}|X^{max}_i\ge K_q]$	0	0.5	1.00676	10.60944
					$\sigma [T_{i,K_q}|X^{max}_i\ge K_q]$	0	0.5	0.82202	6.66161
					$E[N_{i,K_q}|X^{max}_i\ge K_q]$	0	1	2.33784	77.95166
					$\sigma [N_{i,K_q}|X^{max}_i\ge K_q]$	0	0	0.67229	43.81203
	50	1.01919	0.01920	0.01959	$$K_q $$	1	1	1	2
					$\mathbb {\tilde{P}}(X^{max}_i\ge K_q)$	0.99959	0.99959	0.99959	0.01959
					$E[T_{i,K_q}|X^{max}_i\ge K_q]$	0	0	0	0.98000
					$\sigma [T_{i,K_q}|X^{max}_i\ge K_q]$	0	0	0	0.98000
					$E[N_{i,K_q}|X^{max}_i\ge K_q]$	0	0	0	1
					$\sigma [N_{i,K_q}|X^{max}_i\ge K_q]$	0	0	0	0
	100	1.00990	0.00990	0.01000	$$K_q $$	1	1	1	2
					$\mathbb {\tilde{P}}(X^{max}_i\ge K_q)$	0.99990	0.99990	0.99990	0.01000
					$E[T_{i,K_q}|X^{max}_i\ge K_q]$	0	0	0	0.98990
					$\sigma [T_{i,K_q}|X^{max}_i\ge K_q]$	0	0	0	0.98990
					$E[N_{i,K_q}|X^{max}_i\ge K_q]$	0	0	0	1
					$\sigma [N_{i,K_q}|X^{max}_i\ge K_q]$	0	0	0	0

**Table 3:** Expected values $E[X^{max}_i]$, $E[T^{max}_i]$ and $E[N^{max}_i]$ for different choices of $(\nu ,\langle n \rangle )$ in the soft niche case, for the parameters $(\mu ,\varphi )=(1.0,500.0)$ and the initial clonal size $i=1$

$\nu$	$\langle n \rangle$	$E[X^{max}_i]$	$E[T^{max}_i]$	$E[N^{max}_i]$	*q*	0.25	0.5	0.75	0.99
500	1	3.64779	1.36144	9.36164	$$K_q $$	1	1	3	44
					$\mathbb {\tilde{P}}(X^{max}_i\ge K_q)$	0.99048	0.99048	0.32293	0.00066
					$E[T_{i,K_q}|X^{max}_i\ge K_q]$	0	0	1.00166	23.19127
					$\sigma [T_{i,K_q}|X^{max}_i\ge K_q]$	0	0	0.81786	14.13329
					$E[N_{i,K_q}|X^{max}_i\ge K_q]$	0	0	2.33378	363.97348
					$\sigma [N_{i,K_q}|X^{max}_i\ge K_q]$	0	0	0.66722	222.16995
	50	1.01882	0.01884	0.01922	$$K_q $$	1	1	1	2
					$\mathbb {\tilde{P}}(X^{max}_i\ge K_q)$	0.99961	0.99961	0.99961	0.01922
					$E[T_{i,K_q}|X^{max}_i\ge K_q]$	0	0	0	0.98039
					$\sigma [T_{i,K_q}|X^{max}_i\ge K_q]$	0	0	0	0.98039
					$E[N_{i,K_q}|X^{max}_i\ge K_q]$	0	0	0	1
					$\sigma [N_{i,K_q}|X^{max}_i\ge K_q]$	0	0	0	0
	100	1.00970	0.00971	0.00980	$$K_q $$	1	1	1	1
					$\mathbb {\tilde{P}}(X^{max}_i\ge K_q)$	0.99010	0.99010	0.99010	0.99010
					$E[T_{i,K_q}|X^{max}_i\ge K_q]$	0	0	0	0
					$\sigma [T_{i,K_q}|X^{max}_i\ge K_q]$	0	0	0	0
					$E[N_{i,K_q}|X^{max}_i\ge K_q]$	0	0	0	0
					$\sigma [N_{i,K_q}|X^{max}_i\ge K_q]$	0	0	0	0
1000	1	1.54331	0.34871	0.64267	$$K_q $$	1	1	2	6
					$\mathbb {\tilde{P}}(X^{max}_i\ge K_q)$	0.99225	0.99225	0.32536	0.00795
					$E[T_{i,K_q}|X^{max}_i\ge K_q]$	0	0	0.66689	2.77159
					$\sigma [T_{i,K_q}|X^{max}_i\ge K_q]$	0	0	0.66689	1.77604
					$E[N_{i,K_q}|X^{max}_i\ge K_q]$	0	0	1	7.28252
					$\sigma [N_{i,K_q}|X^{max}_i\ge K_q]$	0	0	0	2.52032
	50	1.00970	0.00971	0.00980	$$K_q $$	1	1	1	1
					$\mathbb {\tilde{P}}(X^{max}_i\ge K_q)$	0.99010	0.99010	0.99010	0.99010
					$E[T_{i,K_q}|X^{max}_i\ge K_q]$	0	0	0	0
					$\sigma [T_{i,K_q}|X^{max}_i\ge K_q]$	0	0	0	0
					$E[N_{i,K_q}|X^{max}_i\ge K_q]$	0	0	0	0
					$\sigma [N_{i,K_q}|X^{max}_i\ge K_q]$	0	0	0	0
	100	1.00493	0.00493	0.00495	$$K_q $$	1	1	1	1
					$\mathbb {\tilde{P}}(X^{max}_i\ge K_q)$	0.99503	0.99503	0.99503	0.99503
					$E[T_{i,K_q}|X^{max}_i\ge K_q]$	0	0	0	0
					$\sigma [T_{i,K_q}|X^{max}_i\ge K_q]$	0	0	0	0
					$E[N_{i,K_q}|X^{max}_i\ge K_q]$	0	0	0	0
					$\sigma [N_{i,K_q}|X^{max}_i\ge K_q]$	0	0	0	0

The distribution of $X_i^{max}$ is analysed in Tables 1, 2 and 3 by computing its (100*q*)th percentiles $K_q$ for $q\in \{0.25,0.5,0.75,0.99\}$, where the percentile $K_q$ is the first value $x\ge 1$, such that $\mathbb {P}(X_i^{max}\le x)\ge q$. The probability $\mathbb {P}(X_i^{max}\ge K_q)$ of reaching the clonal sizes represented by $K_q$ can be exactly obtained from Eq. (4). However, these probabilities are replaced in Tables 1, 2 and 3 by their truncated versions ${\mathbb {\tilde{P}}}(X_i^{max}\ge K_q)=\mathbb {P}(K_{0.99}\ge X_i^{max}\ge K_q)$. We have done so as these truncated values result in good approximations to the true probabilities, but with the advantage that, for the particular cases yielding $K_q=1$ (for example, $\varphi \in \{0.1,1.0\}$ and $q=0.25$ in Table 1), they provide the total probability mass of $X_i^{max}$ considered in the truncations given by Eqs. (7) and (10).

The time and the number of proliferation events to reach the maximum clonal size can be analysed in greater depth by studying the conditional values $E[T_{i,K_q}|X^{max}_i\ge K_q]$, $\sigma [T_{i,K_q}|X^{max}_i\ge K_q]$, $E[N_{i,K_q}|X^{max}_i\ge K_q]$ and $\sigma [N_{i,K_q}|X^{max}_i\ge K_q]$, where $\sigma [X]$ denotes the standard deviation of the random variable *X*. We note here that we consider conditional values due to the fact that the random variables $T_{i,K_q}$ and $N_{i,K_q}$ are defective; that is, we have $T_{i,K_q}=N_{i,K_q}=\infty$ if the clonal size $K_q$ is not reached, which occurs with non-zero probability. These values provide information about the time and the number of proliferation events to reach different clonal sizes ($K_q$ with $q\in \{0.25,0.5,0.75,0.99\}$), under the assumption that those clonal sizes have been, in fact, reached.

When focusing on the hard niche case (Table 1; Fig. 3), the scenarios that allow the establishment of the RTE in the periphery are given by values of $E[X_i^{max}]$ and $E[T_i^{max}]$ significantly larger than 1, which correspond to larger values of the homeostatic signalling rate $\varphi$. It is clear that the mean value, $E[X^{max}_i]$, has a clearly increasing behaviour with respect to the signalling rate $\varphi$. Moreover, results corresponding to those hostile environments within the hard niche case, corresponding to low signal rates, represent the pressure against the survival and expansion of the clonotype in the short-term. In those cases ($\varphi \in \{0.1,1.0,5.0\}$), the most representative sample path is the one which amounts to the immediate extinction of the clonotype from the initial clonal size $i=1$, which gives $X_i^{max}=1$, or the occurrence of a few proliferation events (yielding a maximum size of the clonotype near 1), and the subsequent extinction of the clonotype. On the other hand, in those cases where the clonotype survives in the mid-term (higher signalling rates), we observe a high concentration of the probability mass function of $X_i^{max}$ around some mode, which is reflected in the concentrated values obtained for the percentiles $K_{0.25}$, $K_{0.5}$, $K_{0.75}$ and $K_{0.99}$. In particular, the probability mass function of $X_i^{max}$ is unimodal when the clonotype becomes extinct in the short-term with high probability, and bimodal when the clonotype survives and expands in the mid-term, with moderate probabilities.

The bimodal shape of $X_i^{max}$ in friendly, yet competitive peripheral environments, within the hard niche case, becomes more apparent when analysing results plotted in Fig. 3. In particular, we plot in Fig. 3 the probability mass function of $X_i^{max}$ for the hard niche case for signalling rates $\varphi \in \{0.1,1.0,5.0,10.0\}$. The clonotype under consideration has higher potential for expansion for larger values of the homeostatic signalling rate $\varphi$. However, in these cases, the probability mass function of $X_i^{max}$ shows a bimodal shape, with a mode equal to 1 and the other taking different values depending on the particular parameters. This shape, together with the results of Table 1, should be interpreted as a binary outcome scenario: in these cases, the clonotype has moderate or high probabilities of reaching its potential clonal size represented by the second mode, which will only happen if it escapes the extinction at the beginning of its lifetime; on the other hand, there exists, with some significant probability, the chance of the clonotype becoming extinct in the short-term (during its first transitions), which yields the first mode equal to 1. We point out here that this also means that (for example, see the case $\varphi =10.0$ in Fig. 3), once the clonotype escapes extinction in the short-term, the probabilities of reaching different clonal sizes, such as $i_{\varrho }\in \{5,10,15\}$, are practically equal, since they represent the probability of reaching clonal sizes around the second mode. Finally, when the rate of signal is not enough (see the case $\varphi =0.1$ in Fig. 3), the probability mass function of $X_i^{max}$ is unimodal around 1. This represents the case in which the clonotype will become immediately extinct (or extinct in the short-term after reaching small sizes around 1) from its initial clonal size 1.

The increase of $E[X_i^{max}]$ associated with increasing values of the homeostatic signalling rate in Table 1 leads also to increasing mean values for $E[T^{max}_i]$, $E[N^{max}_i]$, $E[T_{i,K_q}|X^{max}_i\ge K_q]$ and $E[N_{i,K_q}|X^{max}_i\ge K_q]$. This is easily explained by noting that, in these cases, the clonotype has a greater potential to reach bigger clonal sizes, so that the time and number of proliferation events to reach those sizes should also be larger. The variability of these random variables increases, as well, which is observed by analysing the values $\sigma [T_{i,K_q}|X^{max}_i\ge K_q]$ and $\sigma [N_{i,K_q}|X^{max}_i\ge K_q]$. Moreover, the coefficient of variance $CV(X)=\sigma [X]/E[X]$ of these random variables seems to slowly increase to one, when increasing $\varphi$. In any case, the large values of $E[T^{max}_i]$ and $E[N^{max}_i]$ imply that, although in friendly conditions a substantial clonotype maximum size will be reached with high probability, there exists a significant impediment against this event, represented by the large number of proliferation and cell death events taking place until this maximum size is reached.

The intermediate niche case is analysed in Table 2 and Fig. 4. In this case, friendly environments can be identified with small values of $\nu$ ($\nu =1$ and $\langle n \rangle \in \{1,50,100\}$ in Table 2), or with moderate values of $\nu$ together with small values of $\langle n \rangle$ ($\nu =10$ and $\langle n \rangle =1$ in Table 2), while hostile scenarios correspond to greater numbers of competitors and larger clonal sizes for them. Again, the clonotype (or RTE) can become established in the periphery under friendly enough environments, reaching a maximum clonal size near $E[X_i^{max}]$, represented by the larger mode of the distribution of $X_i^{max}$ (given by the value around which its percentiles are concentrated). Under too hostile scenarios, the clonotype will not become established and its maximum clonal size remains near 1. Again, this binary outcome scenario can be properly identified by analysing the results in Fig. 4, where the probability mass function of $X_i^{max}$ is plotted for different parameter regimes for the intermediate niche case. It is worth noting that even in the case in which the most probable outcome is the establishment of the clonotype (*e.g.,* $\nu =1$ and $\langle n \rangle =1$ in Fig. 4), there exists a non-negligible probability of extinction in the short-term. This probability is identified with the probability mass of $X_i^{max}$ around the value 1.

We note here that there exist several scenarios where a certain expected behaviour can be identified. For example, in the particular case $(\nu ,\langle n \rangle )=(50,50)$ in Table 2, we obtain the following percentiles of $X_i^{max}$: $(K_{0.25},K_{0.5},K_{0.75},K_{0.99})=(1,1,1,2)$, which represent a high concentration of the probability mass function around the value 1. That is, there is a high probability of the clonotype becoming immediately extinct from its initial clonal size $i=1$. In particular, for percentiles $K_q=1$, the corresponding time $T_{i,K_q}$ to reach the clonal size $K_q$ is equal to 0, since the initial clonal size is already $i=1$, and the standard deviation equals 0. Similar comments can be made for $N_{i,K_q}$. The number of proliferation events to reach $K_q=2$ (given that it is reached) is equal to 1, with probability 1, and then its standard deviation is 0. It should be noted that the distribution of the random time $T_{i,2}$, under the assumption that $T_{i,2}<\infty$, can be seen as the distribution of the minimum between two independent and exponentially distributed random variables *X* and *Y* with means $\lambda _1^{-1}$ and $\mu _1^{-1}$ in the case $X<Y$. As a result, $T_{i,2}$ is exponentially distributed with mean $(\lambda _1+\mu _1)^{-1}$, and $E[T_{i,2}|X^{max}_i\ge 2]=\sigma [T_{i,2}|X^{max}_i\ge 2]=1/(\lambda _1+\mu _1)=0.98000$. Moreover, we note that one should carefully interpret the value of $E[X_i^{max}]$ in Tables 1, 2 and 3, since these values have been obtained making use of the truncation procedure described in Eqs. (7) and (10).

Finally, the soft niche case representing a hostile environment given by a large number of competitors and big clonal sizes for them, is analysed in Table 3 and Fig. 5. Results in Table 3 represent scenarios where the most probable outcome is the short-term extinction of the clonotype (values of $E[X_i^{max}]$, $E[T_i^{max}]$ and $E[N_i^{max}]$ near 1, percentiles $K_q$ accumulated around 1). This outcome can be identified by analysing the probability mass function of $X_i^{max}$ for these cases, which is concentrated around 1 (see Fig. 5).

**Fig. 6:**
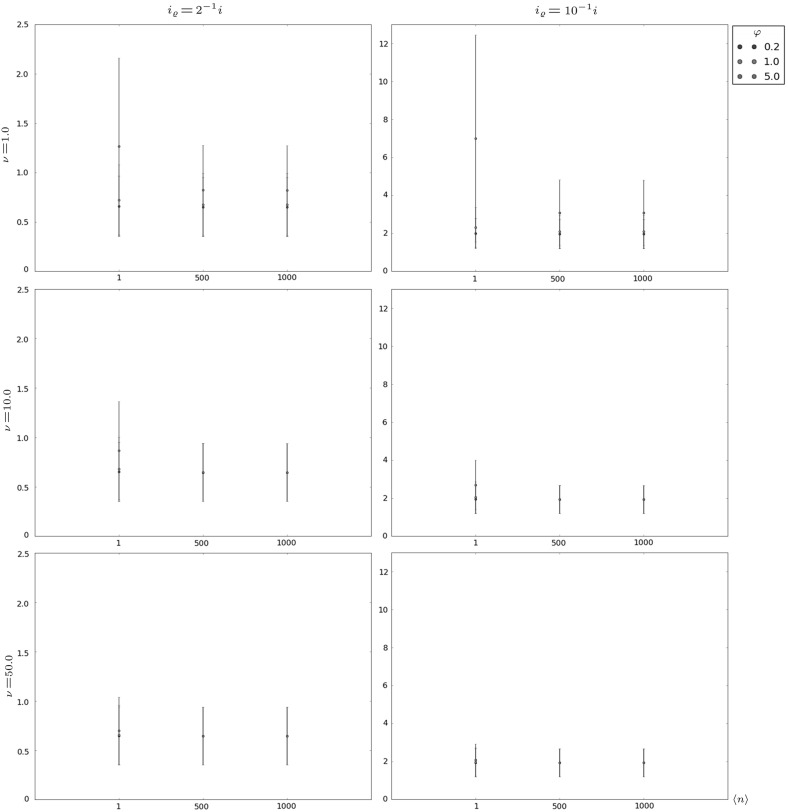
Expected value $E[T_{i,i_{\varrho }}]$ plus and minus standard deviation $\sigma [T_{i,i_{\varrho }}]$ for $i=10$, in the intermediate niche case, for $\mu =1.0$ and various choices of $i_{\varrho }$, $\nu$, $\varphi$ and $\langle n \rangle$

**Fig. 7:**
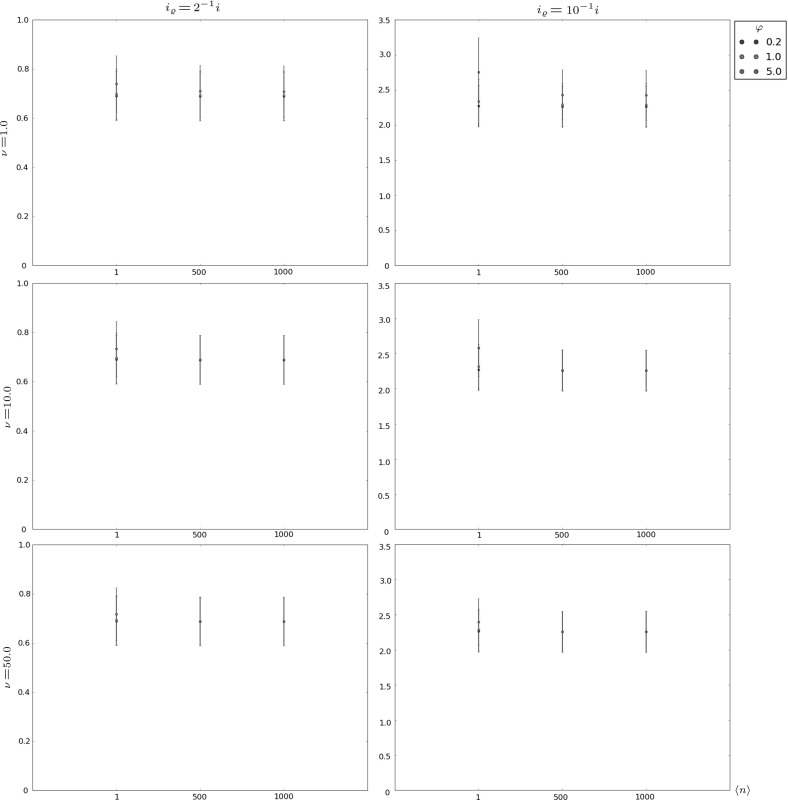
Expected value $E[T_{i,i_{\varrho }}]$ plus and minus standard deviation $\sigma [T_{i,i_{\varrho }}]$ for $i=100$, in the intermediate niche case, for $\mu =1.0$ and various choices of $i_{\varrho }$, $\nu$, $\varphi$ and $\langle n \rangle$

**Fig. 8:**
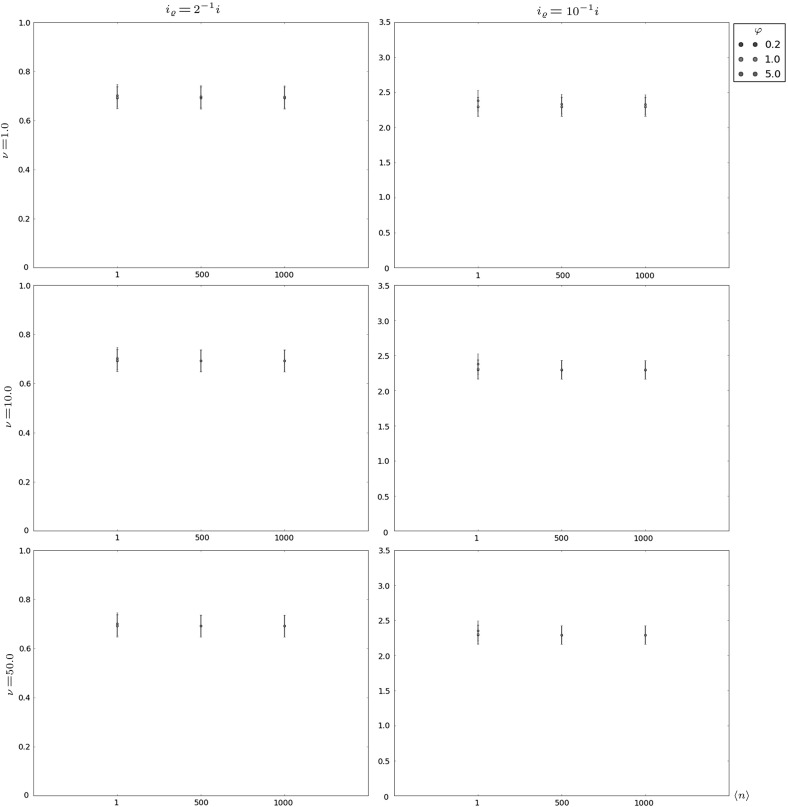
Expected value $E[T_{i,i_{\varrho }}]$ plus and minus standard deviation $\sigma [T_{i,i_{\varrho }}]$ for $i=500$, in the intermediate niche case, for $\mu =1.0$ and various choices of $i_{\varrho }$, $\nu$, $\varphi$ and $\langle n \rangle$

**Fig. 9:**
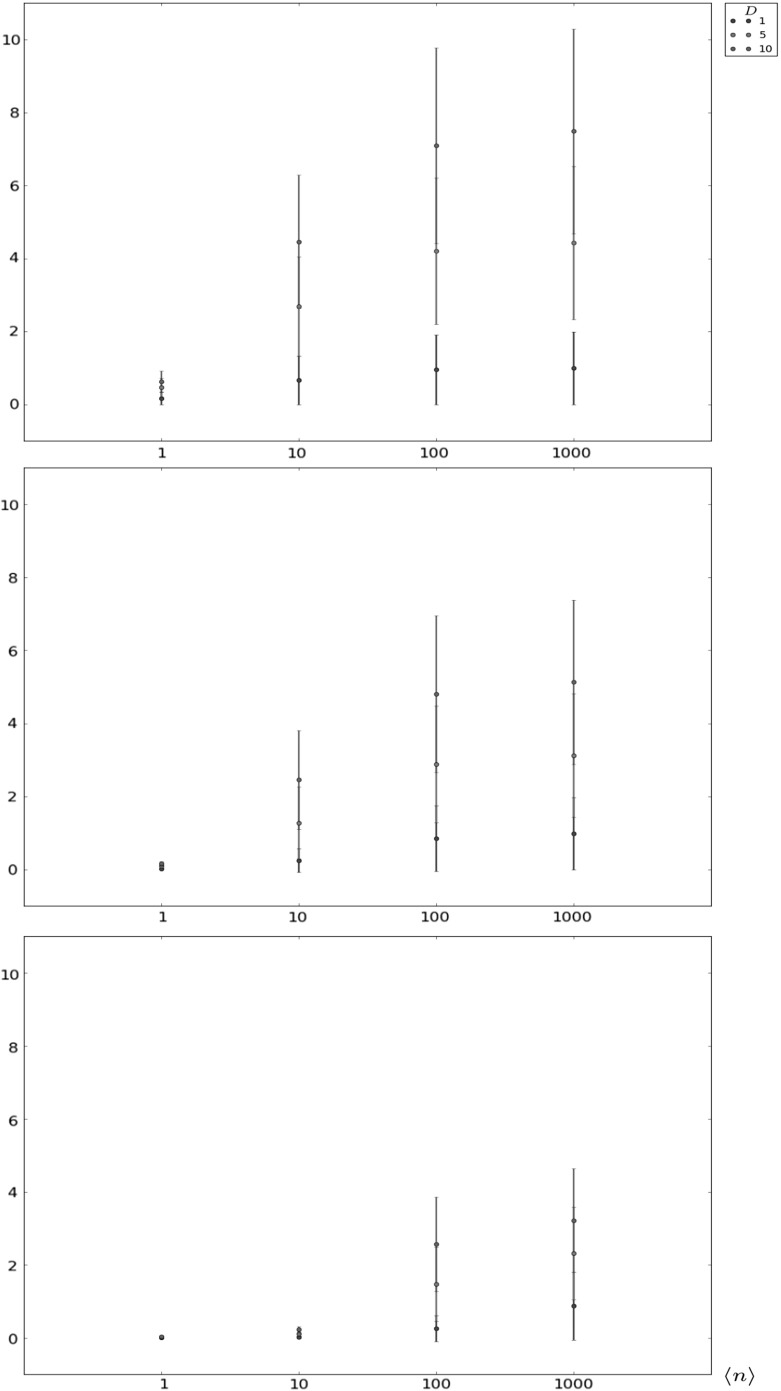
Conditional expected value $E[T_i^D|T_i^D<\infty ]$ plus and minus conditional standard deviation $\sigma [T_i^D|T_i^D<\infty ]$ for (from *top* to *bottom*) $i\in \{1,10,100\}$, in the soft niche case, and for various choices of the number *D* of proliferation events, and the parameters $\mu =1.0$, $\varphi =1000.0$ and $\nu =200.0$

In Figs. 6, 7, 8 and 9 and Table 4, we focus on the auxiliary descriptors $T_{i,i_{\varrho }}$, with $i_{\varrho }<i$, and $T_i^D$, which have not been analysed previously. In particular, we consider in Figs. 6, 7, and 8 the intermediate niche case and we analyse the time $T_{i,i_{\varrho }}$ to contraction to a given clonal size $i_{\varrho }<i$. The mean time, $E[T_{i,i_{\varrho }}]$, and the standard deviation, $\sigma [T_{i,i_{\varrho }}]$, are computed for different values of *i* ($i=10$, 100 and 500 for Figs. 6, 7 and 8, respectively), $i_{\varrho }$, and parameters $\nu$, $\varphi$ and $\langle n \rangle$. The results in these figures show relatively little dependence of this time to contraction on the competition parameters ($\nu ,\langle n \rangle$). The initial *i* and final $i_{\varrho }$ clonal sizes, and the signalling rate $\varphi$ seem to have a higher impact on the behaviour of this random variable, since larger values of $\varphi$ are related to a higher resistance of the clonotype against contraction.

In Table 4 and Fig. 9, the time $T_i^D$ to reach a particular number of proliferation events, *D*, is studied for the soft niche case. In particular, the probabilities $\mathbb {P}(T_i^D<\infty )$ are given in Table 4, and the conditional mean values $E[T_i^D|T_i^D<\infty ]$ and conditional standard deviations $\sigma [T_i^D|T_i^D<\infty ]$ are shown in Fig. 9 for different values of *i*, *D* and $\langle n \rangle$. In contrast to the time to contraction, the results of Table 4 and Fig. 9 indicate that $T_i^D$ (which is a measure of the proliferation capacity of the clonotype) seems to be highly sensitive to $\langle n \rangle$, the characteristic number of naive T cells in a competitor clonotype. When the competition environment is too hostile, which is represented by large values of $\langle n \rangle$, the potential of the clonotype in the periphery to reach a certain number, *D*, of proliferation events is notably reduced, and this is reflected by the decrease of the probabilities $\mathbb {P}(T_i^D<\infty )$. At the same time, means and standard deviations in Fig. 9 increase in those scenarios. On the other hand, for small values of $\langle n \rangle$, there exist parameter regimes where the target number of divisions, *D*, is easily reached (see, for example, the cases associated with the initial clonal size $i=100$ in Table 4).

## Discussion

In this paper we have analysed the fate of a given naive T cell clonotype or a recent thymic emigrant (with initial clonal size $i=1$), when competing with pre-established peripheral T cell clonotypes for homeostatic proliferation signals. The dynamics of a given clonotype has been based on a mathematical stochastic model originally developed in Stirk et al. (2008). Here, our objective is to study the fate and potential of naive T cell clonotypes in the periphery by analysing, for a given clonotype, its maximum clonal size, the time to reach this maximum, the number of proliferation events required to reach this maximum, the rate of contraction of the clonotype during its way to extinction, as well as the time to a given number of proliferation events.

We have introduced in Sect. 2 several stochastic descriptors in terms of defective and non-defective random variables. The use of Laplace-Stieltjes transforms, probability generating functions, auxiliary random variables and absorbing augmented Markov processes, together with first-step analysis, allow us to obtain, in some cases, the distribution of the random variables of interest, or to exactly compute or approximate the different order moments of these variables.

**Table 4:** Probabilitiy $\mathbb {P}(T_i^D<\infty )$ as a function of the initial clonal size *i* in the soft niche case, for various choices of the number *D* of proliferation events, and the parameters $\mu =1.0$, $\varphi =1000.0$ and $\nu =200.0$

*i*	*D*	$\langle n \rangle$	1	10	100	1000
1	1		0.83263	0.33322	0.04761	0.00497
	5		0.79944	0.05986	${<}10^{-5}$	${<}10^{-9}$
	10		0.79876	0.01653	${<}10^{-9}$	${<}10^{-18}$
10	1		0.99999	0.98249	0.38600	0.04865
	5		0.99999	0.74816	0.00113	${<}10^{-7}$
	10		0.99999	0.41791	${<}10^{-6}$	${<}10^{-16}$
100	1		1	1	0.99230	0.39263
	5		1	0.99999	0.58648	0.00022
	10		1	0.99999	0.05467	${<}10^{-9}$

In Sect. 3 several numerical experiments have been carried out in order to study the descriptors previously defined, under different environmental conditions for the clonotype under consideration. In particular, when a recent thymic emigrant reaches the periphery, it will compete with a small or large number of different clonotypes depending on the TCR it expresses (which in turn determines its cross-reactivity). The parameter that encodes the number of competitors is $\nu$ in the model. The characteristic clonal size of these competitors, $\langle n \rangle$, as well as the total rate of stimulatory signal provided by the self-peptides that the given clonotype can recognise, $\varphi$, also affect the dynamics of the clonotype under study, so that three different regimes are defined (hard, soft or intermediate niche). For these three different scenarios, numerical results have been provided for our descriptors, which allow us to follow the survival and extinction dynamics of the clonotype or RTE. Our main results can be summarised as follows:

1. Two fates can be identified for the dynamics of the clonotype: if the clonotype under study does not receive enough stimulatory signal, or if it faces too hostile a competitive environment (encoded by a large number of competitors and/or large clonal sizes of these competitors), the survival probability of the clonotype in the mid-term is low. This fate can be seen by analysing the results in Fig. 4 for $\nu =50$, or Fig. 5. If the clonotype faces moderate competitive environments, the probability of the clonotype escaping extinction and surviving in the long-term is significant, and it can be approximated in terms of the probability mass function of $X_i^{max}$.
2. In this second case, the bimodal structure of the probability mass function of $X_i^{max}$ (where one mode is 1 or near 1, and the other mode is far away from it) reflects a binary outcome scenario:
  One possibility is the extinction of the clonotype in the short-term, with non-negligible probability. The probability of this event can be identified with the sum of the probabilities around 1 in the probability mass function of $X_i^{max}$.
  If such an event does not occur, the clonotype escapes extinction and it reaches its maximum size, which will be a value near the second mode of the probability mass function of $X_i^{max}$.
  For example, in Fig. 4 for $\nu =1$ and $\langle n \rangle =1$, the probability mass around 1 represents the probability of the clonotype becoming extinct after a few proliferation and death events (then, the maximum clonal size will be 1 or a value near 1). The long term survival probability of the clonotype can be approximately identified with $\mathbb {P}(X_i^{max}\ge 6)$. We note here that the probability mass for states between 6 and 110 is almost null, which reflects the fact that once the clonotype escapes extinction, it will reach maximum clonal sizes above 110 almost surely. Moreover, the high concentration of the probability mass function around the second mode (which can also been identified by analysing the percentiles of $X_i^{max}$ in Tables 1, 2 and 3) should be interpreted as the fact that the set of parameters $(\varphi ,\nu ,\langle n \rangle )$ (and, thus, the molecular properties of the corresponding TCR and the clonal sizes of competing T cell clonotypes) highly determines the maximum size of a clonotype in the periphery.

Recent experimental work suggests that whether a recent thymic emigrant survives or not upon its arrival in the periphery, depends on the clonal sizes of the pre-existing and competing naive T cell clonotypes: under lymphopenic conditions, that is, small values of $\langle n \rangle$, RTEs seem to be able to proliferate and become established in the periphery, but when the peripheral naive T cell pool is full, RTEs struggle to get incorporated into the population of recirculating lymphocytes (Fink and Hendricks 2011; Houston et al. 2011; Berkley and Fink 2014). This empirical observation is in line with our results: the fate of a recent thymic emigrant during its initial journey in the periphery has a clear stochastic component, where the probability of extinction cannot be neglected, even under favourable conditions for the RTE, and which can be studied in terms of the mass probability function of $X_i^{max}$. On the other hand, a greater deterministic component can be identified in the potential size of the clonotype seeded by the RTE in the long-term, once it escapes extinction (which is more likely for small values of $\langle n \rangle$), which is reflected in the concentration of the mass probability function of $X_i^{max}$ around the second mode.

Other interesting results that have been derived from our study of the stochastic descriptors defined here are:

1. There seems to exist some threshold value for the parameter $\varphi$ when analysing the time and the number of proliferation events to reach the maximum clonal size. This threshold behaviour can be observed, for example, in Table 1 for parameters $\varphi \in \{10.0,20.0\}$ and $\nu =0$ (hard niche case). It can be observed that, for these cases, small changes in $E[X_1^{max}]$ are, however, associated with large changes in $E[T_1^{max}]$ and $E[N_1^{max}]$. These results suggest that different clonotypes with similar maximum clonal sizes can display very different behaviours as to how this maximum size is achieved (average time and number of divisions), depending on the value of $\varphi$, the homeostatic proliferation signalling rate.
2. Figures 6, 7 and 8 allow us to show that the rate of contraction of the given clonotype on its way to extinction seems to depend much more on $\varphi$, the homeostatic proliferation signalling rate, than on the competition parameters $(\nu ,\langle n \rangle )$. At the same time, the competition parameters do significantly affect the proliferation rate of the clonotype, which is analysed in Table 4 and Fig. 9, in terms of the time to reach a particular number of proliferation events.
